# Band gap engineering in Al, Cu, N and Al/Cu Co-doped ZnO thin films: experimental study and quantum machine learning prediction

**DOI:** 10.1038/s41598-026-55151-2

**Published:** 2026-05-25

**Authors:** Amir Hossein Salehi Shayegan, Laya Dejam

**Affiliations:** 1 Faculty of Mathematics, K. N. Toosi University of Technology, Tehran, Iran; 2 Physics Department, West Tehran Branch, Islamic Azad University, Tehran, Iran

**Keywords:** ZnO thin films, RF magnetron sputtering, Band-gap engineering, Quantum machine learning, Transparent conductive oxides, Materials science, Nanoscience and technology, Optics and photonics, Physics

## Abstract

This study investigates RF magnetron sputtered ZnO thin films doped with Al, Cu, N and co-doped with Al/Cu, followed by post deposition annealing at 300-600 °C. Structural and compositional characterization was performed by X ray diffraction (XRD) and energy dispersive spectroscopy (EDS), while surface morphology and roughness were assessed by atomic force microscopy (AFM). Optical band gaps were extracted experimentally from UV-Vis spectra using Tauc analysis and Urbach energy evaluation. Key descriptors, lattice constants, crystallite size, root mean square (RMS) roughness, and Urbach energy were used as input features for a data driven quantum machine learning model. A variational quantum regression (VQR) model trained on these structural and processing descriptors achieved a mean squared error (MSE) of 0.0576 eV in predicting band gap values, accurately capturing trends induced by dopant type and annealing temperature. By combining systematic experimental tuning with quantumn enhanced predictive modeling, the work provides an integrated framework for accelerated band gap engineering of ZnO thin films, with direct implications for optimizing transparent conductive oxides and other optoelectronic materials.

## Introduction

Zinc oxide (ZnO), a II–VI compound semiconductor crystallizing in the hexagonal wurtzite structure, is a key transparent conductive oxide (TCO) for optoelectronic applications. With a direct bandgap of approximately $E_g \approx 3.37$ eV and a high exciton binding energy (*∼ 60* meV), ZnO is intrinsically suited for transparent electrodes, ultraviolet emitters, and thin-film transistors. The incorporation of aliovalent and isovalent dopants provides a fundamental mechanism for tuning carrier density, mobility, and defect equilibria, thereby enabling optimization of the conductivity-transparency figure of merit. Recent investigations employing RF magnetron sputtering have shown that dopant species, concentration gradients, and deposition kinetics critically influence microstructural evolution, optical bandgap shifts, and defect-mediated absorption. These findings highlight the necessity of rigorous comparative analyses across distinct doping regimes^1^.

Among physical vapor deposition (PVD) techniques, RF magnetron sputtering is widely recognized for synthesizing transparent conductive oxides (TCOs). It offers industrial scalability, reproducibility, and precise control of stoichiometry through adjustments in target composition, RF power density, and working pressure. Careful tuning of $\mathrm{Ar/O}_2$/$\textrm{N}_2$ gas flow ratios and discharge power enables modulation of film packing density, crystallographic texture-particularly the preferred c-axis orientation-and dopant incorporation efficiency. These parameters directly influence the optoelectronic performance of the resulting films^2^. In the present study, single-doped (Al, Cu, N) and co-doped (Al/Cu) ZnO thin films were deposited on quartz substrates and subjected to post-annealing across a range of 300-600 °C. This thermal processing was utilized to systematically probe the kinetics of defect recovery, grain coarsening and dopant activation as a function of thermal treatment.

The incorporation of N and Cu dopants in ZnO was strategically designed to enable defect-state engineering and band structure modulation beyond the conventional Al-doped ZnO (AZO) framework. While AZO chiefly increases n-type conductivity by substituting $\mathrm{{A}}{\mathrm{{l}}^{\mathrm{{3 + }}}}$ for $\mathrm{{Z}}{\mathrm{{n}}^{\mathrm{{2 + }}}}$, it offers limited control over intrinsic defect chemistry and frequently produces Burstein-Moss band widening when free-carrier concentrations become excessive.

In contrast, nitrogen doping is introduced to promote acceptor-like behavior through substitution at oxygen sites (${\mathrm{{N}}_\mathrm{{O}}}$), thereby compensating for intrinsic donor defects such as oxygen vacancies (${\mathrm{{V}}_\mathrm{{O}}}$) and zinc interstitials ($\mathrm{{Z}}{\mathrm{{n}}_\mathrm{{i}}}$). This compensation mechanism reduces unintentional n-type conductivity and enables controlled modification of states near the valence band edge.

Similarly, Cu doping serves as a dual electronic function. When substituting Zn sites, Cu can introduce acceptor-related levels, while the interaction between Cu 3d and O 2p orbitals modifies the valence band structure through p-d hybridization. This interaction facilitates band gap tuning without relying solely on carrier-density-induced effects. Additionally, Cu incorporation influences defect equilibria and microstructure, further affecting optical absorption and interfacial charge transfer characteristics.

Unlike conventional AZO systems, where Al doping primarily aims to increase free carrier concentration and electrical conductivity, the purpose of N and Cu doping in ZnO is broader and mainly focused on defect and electronic structure engineering. Nitrogen incorporation is intended to introduce acceptor-like states and partially compensate the intrinsic n-type character of ZnO, thereby modifying defect-related band tail states and optical transitions rather than solely achieving stable p-type conductivity. In parallel, Cu doping is used to tailor localized electronic states, defect-assisted recombination, and interfacial charge transfer characteristics through the formation of intermediate energy levels and modified grain-boundary properties.

Also, unlike AZO systems that focus predominantly on conductivity enhancement, N and Cu doping provide a multidimensional strategy involving carrier compensation, defect engineering, and band alignment control. This enables more precise band gap engineering and improved interfacial property optimization, which are critical for advanced optoelectronic, transparent electronics, and photovoltaic device architectures.

The dopants were selected to probe distinct and complementary defect physics within the ZnO lattice. Aluminium substitutes for zinc (Al_Zn_) as a shallow donor with an ionization energy of approximately 50-120 meV, thereby elevating the free-electron concentration and inducing a Burstein-Moss shift of the optical absorption edge toward higher energies at elevated doping levels. Copper, depending on its oxidation state and lattice site occupancy (Cu_Zn_ or Cu_i_), introduces localized *d*-states within the forbidden gap that act as deep acceptors or recombination centers, modifying both carrier mobility and minority-carrier lifetime ^3^. Nitrogen substitution on oxygen sites (N_O_) has been proposed as a candidate for *p*-type doping; however, its high ionization energy and propensity to form compensating complexes (e.g., N_O_-V_O_) often result in persistent *n*-type behavior while simultaneously affecting sub-bandgap absorption through defect-mediated transitions. Al/Cu co-doping was investigated to assess potential donor-acceptor compensation, charge-transfer interactions and their collective influence on free-carrier screening and mid-gap state density. Such co-doping strategies may enable independent optimization of electrical conductivity and optical transparency by balancing shallow donor contributions against deep-level recombination.

Structural characterization was performed using X-ray diffraction (XRD) to determine phase purity, crystallite size via the Scherrer equation and residual lattice strain from peak broadening analysis. Elemental composition and spatial homogeneity were verified by energy-dispersive X-ray spectroscopy (EDS), while surface morphology and root-mean-square roughness were quantified by atomic force microscopy (AFM). Optical properties were probed by UV-Vis spectrophotometry over the 200-2500 nm range. Direct optical bandgaps ($E_\textrm{g}$) were extracted by linear extrapolation of $(\alpha h\nu )^2$ versus photon energy (*hν*) plots according to the Tauc relation for direct transitions. The Urbach energy ($E_\textrm{U}$), a measure of band-tail disorder arising from structural and compositional fluctuations, was determined from the exponential dependence of the absorption coefficient below the band edge. These analyses were used to determine the lattice parameters, crystallite size, root-mean-square (RMS) surface roughness, and Urbach energy, which serve as descriptors for predicting the optical band gap of the thin films.

To complement experimental characterization, this study implements a quantum machine-learning (QML) framework for predictive modeling of optical bandgaps. This descriptor set was designed to capture the principal physicochemical factors governing electronic structure while maintaining interpretability and minimizing multicollinearity. The QML architecture integrates classical ensemble methods with quantum feature encoding via parameterized quantum circuits. Variational quantum circuits were employed to map classical descriptors into a high-dimensional Hilbert space, enabling the model to capture complex, nonlinear correlations between processing conditions, microstructural disorder and optical response that may be inaccessible to purely classical regressors. Model hyperparameters were optimized through Bayesian search over a predefined parameter grid.

Predictive performance was rigorously evaluated using *k*-fold cross-validation (*k = 5*) with stratification by dopant type to ensure representative sampling across all doping conditions. Generalization capability was further assessed by reserving specific annealing temperatures as held-out test sets, simulating the practical scenario of predicting bandgaps for untested thermal treatments. Model accuracy was quantified using root-mean-square error (RMSE), mean absolute error (MAE) and the coefficient of determination ($R^2$).

The objectives of this work are threefold: quantify how Al, Cu, N and Al/Cu co-doping shift the optical bandgap and Urbach energy across the annealing temperature range; correlate microstructural evolution and defect signatures with observed optical changes; and demonstrate the predictive accuracy of the quantum-ML model while extracting interpretable feature importances.

Optical band gap prediction in ZnO thin films has traditionally relied on classical machine learning approaches such as Gaussian Process Regression (GPR), Artificial Neural Networks (ANN), and Kernel Ridge Regression (KRR), using descriptors including lattice parameters, grain size, and temperature variations. Previous studies, however, have applied these models with limited scope. For instance, Regonia et al. focused solely on the influence of temperature and time in undoped ZnO^4^, while Zhang et al. restricted their analysis to lattice parameters and grain size in doped ZnO systems^5^ . Moreover, the datasets employed in these works were compiled from disparate literature sources, meaning that the samples did not share consistent growth conditions, and the effects of varying annealing temperatures were not systematically incorporated.

Previous studies have compared experimentally measured and theoretically calculated band gaps, often focusing on undoped ZnO^6^ . In contrast, our objective is to fabricate ZnO thin films under systematically varied deposition temperatures and dopant conditions, and to deliberately engineer their structural and optical properties. At the same time, advances in quantum computing and quantum machine learning open new opportunities to model and optimize the processing structure property relationships of widely used thin films. By combining targeted synthesis with quantum enhanced predictive tools, we aim to accelerate materials optimization, improve manufacturing precision, and reduce costly trial and error in device fabrication.

Potential limitations, including dopant solubility constraints, secondary-phase formation and sensitivity of optical fits to scattering and thickness variations, are addressed through complementary characterization and rigorous baseline correction. Together, the integrated experimental and predictive framework presented here offers a pathway toward accelerated bandgap engineering of ZnO-based TCOs and functional oxide thin films for next-generation optoelectronic devices.

This paper is organized as follows. Section Introduction provides the introduction and motivation, Sect. “ Experimental details” details the experimental methods and sample preparation, Sect. “Quantum machine learning framework” describes the quantum machine learning framework and feature selection, Sect. “Results and discussion” presents the results and discussion, with Subsect. “Experimental results” reporting the experimental characterization and Subsect. “Quantum machine learning results” presenting the quantum machine learning predictions and analysis. The paper concludes with a summary of the main findings and perspectives for future work.

## Experimental details

Quartz substrates (10 mm *×* 20 mm) were selected for their optical transparency in the UV–Vis range and thermal stability at elevated annealing temperatures. Prior to deposition, substrates were subjected to a sequential ultrasonic cleaning protocol: immersion in acetone for 15 min to remove organic contaminants, followed by isopropyl alcohol for 15 min to eliminate residual solvent and finally rinsing with deionized water. Cleaned substrates were dried under a nitrogen stream and stored in a dust-free environment until use.

Undoped and doped ZnO thin films were deposited by radio-frequency (RF) magnetron sputtering. A high-purity ZnO target (99.999%) was employed for the growth of undoped ZnO and nitrogen-doped ZnO (N:ZnO) films. For metal-doped variants, custom alloy targets (99.99% purity) with nominal compositions of Zn_0.90_Al_0.10_, Zn_0.90_Cu_0.10_ and Zn_0.90_Al_0.05_Cu_0.05_ (atomic fraction) were prepared by melting the constituent metals at 600 °C and casting into disks (77 mm diameter, 3 mm thickness). The sputtering chamber was evacuated to a base pressure of $10^{-4}$-$10^{-5}$ mbar prior to deposition. The working gas composition was varied according to the dopant species: pure argon (Ar, 99.999%) for undoped ZnO; a mixture of 70% Ar and 30% N_2_ for N:ZnO to provide reactive nitrogen incorporation; and 70% Ar with 30% O_2_ for Al:ZnO, Cu:ZnO, and Al/Cu:ZnO to maintain oxygen stoichiometry during metal-doped film growth. RF power, working pressure and deposition time were optimized independently for each film composition to achieve comparable thicknesses of approximately 230-300 nm. Complete deposition parameters are summarized in Table 1.

**Table 1:** RF magnetron sputtering parameters for undoped and doped ZnO thin films.

Sample	Base pressure (mbar)	Working pressure (mbar)	RF power (W)	Time (min)	Gas composition	Thickness (nm)
ZnO	$2 \times 10^{-4}$	$7.8 \times 10^{-3}$	125	60	100% Ar	230
N:ZnO	$1.4 \times 10^{-4}$	$6.5 \times 10^{-3}$	110	60	70% Ar / 30% N_2_	300
Al:ZnO	$2 \times 10^{-5}$	$6 \times 10^{-3}$	180	115	70% Ar / 30% O_2_	230
Cu:ZnO	$2 \times 10^{-5}$	$6 \times 10^{-3}$	170	70	70% Ar / 30% O_2_	230
Al/Cu:ZnO	$2 \times 10^{-5}$	$6 \times 10^{-3}$	185	78	70% Ar / 30% O_2_	230

Following deposition, samples were subjected to thermal annealing in ambient atmosphere to promote crystallization, defect recovery, and dopant activation. Annealing was performed in a furnace at temperatures of 300, 400, 500, and 600 °C (note: 300 °C was applied only to N:ZnO samples). The heating rate was maintained at 10 °C/min, with a dwell time of 60 min at the target temperature. Samples were subsequently cooled to room temperature at a controlled rate within the furnace to minimize thermal shock and prevent crack formation.

The crystallographic structure and phase purity of the deposited films were analyzed by X-ray diffraction (XRD) using a Philips PW3710 diffractometer equipped with Cu-K*α* radiation (*λ = 0.15406* nm). Diffraction patterns were recorded over the *2θ* range of 10-90° with a step size of 0.02° and a counting time of 2 s per step. Phase identification was performed using X’Pert HighScore software with reference to the ICDD PDF-4+ database.

Elemental composition and dopant concentration were determined by energy-dispersive X-ray spectroscopy (EDS) using a TESCAN VEGA LMU scanning electron microscope operated at an accelerating voltage of 20 kV. Quantitative analysis was performed using standardless ZAF correction to obtain weight and atomic percentages of Zn, O, Al, Cu and N. Multiple point analyses and area scans were conducted to assess compositional homogeneity across the film surface.

Surface topography and grain structure were investigated by atomic force microscopy (AFM) using a Park Scientific Instruments CP AutoProbe operated in contact mode. Scan areas of 1 *μ*m *×* 1 *μ*m and 5 *μ*m *×* 5 *μ*m were acquired with a resolution of 256 *×* 256 pixels. Root-mean-square (RMS) surface roughness ($R_q$) and average grain size were extracted using WSxM image processing software. The evolution of grain morphology and surface roughness as a function of annealing temperature was systematically examined for each doping condition.

Optical transmittance and reflectance spectra were acquired using a Varian Cary 500 Scan UV-Vis-NIR spectrophotometer over the wavelength range of 200-2500 nm. All measurements were performed at room temperature with a bare quartz substrate as the reference for baseline correction.

## Quantum machine learning framework

Quantum Machine Learning (QML) combines quantum feature embeddings with parameterized quantum circuits (PQCs) to approximate nonlinear functions in high-dimensional Hilbert spaces. For a regression task, such as predicting optical band gaps, a QML model consists of a feature map $U_{\phi }(x)$, a variational ansatz *U(θ )* and a measurement observable *M*. Given an input vector $x \in \mathbb {R}^d$, the model prediction is:

1 $$\begin{aligned} f(x;\theta ) = \langle 0 | U_{\phi }^{\dagger }(x)\, U^{\dagger }(\theta )\, M\, U(\theta )\, U_{\phi }(x) | 0 \rangle . \end{aligned}$$

### Quantum feature maps

Quantum feature maps encode classical data into quantum states through unitary transformations:

2 $$\begin{aligned} |\psi (x)\rangle = U_{\phi }(x)\, |0\rangle . \end{aligned}$$

This induces a quantum kernel:

3 $$\begin{aligned} K(x, x') = |\langle \psi (x) | \psi (x') \rangle |^2, \end{aligned}$$

potentially providing an advantage when the mapping is classically intractable.

### ZZFeatureMap

The ZZFeatureMap is a widely used and expressive quantum embedding strategy for quantum machine learning models^7, 8, 9^. It encodes classical data into an *n*-qubit Hilbert space, where the number of qubits is chosen to match the number of input features. Thus, for a feature vector $x = (x_1, \ldots , x_n)$, each feature is mapped to one qubit, establishing a one-to-one correspondence between classical inputs and quantum degrees of freedom. Formally, the feature map is defined as:

4 $$\begin{aligned} U_{\textrm{ZZ}}(x) = \left( \prod _{i<j} e^{i\, \gamma \, x_i x_j\, Z_i \otimes Z_j} \right) \left( \prod _{k=1}^{n} e^{i\, \beta \, x_k\, Z_k} \right) , \end{aligned}$$

where $Z_k$ denotes the Pauli-*Z* operator acting on qubit *k* and $\beta , \gamma$ are hyperparameters (often set to 1 in practice). The structure of $U_{\textrm{ZZ}}(x)$ consists of two components:

1. *Linear Single-Qubit Embeddings.* Each individual feature is encoded through a single-qubit *Z*-rotation:
  5 $$\begin{aligned} R_Z(x_k) = e^{i x_k Z_k}. \end{aligned}$$
  This transformation maps the classical value $x_k$ to a phase rotation on the Bloch sphere of the corresponding qubit. These rotations introduce linear dependence of the quantum state on the input features.
2. *Nonlinear Pairwise Feature Interactions.* Beyond linear encoding, the ZZFeatureMap incorporates entangling two-qubit operations:
  6 $$\begin{aligned} e^{i\, x_i x_j Z_i Z_j}, \end{aligned}$$
  applied for all feature pairs (*i*, *j*) with *i<j*. These gates introduce nonlinear correlations between features by coupling qubits *i* and *j* proportionally to the product $x_i x_j$.

This term is crucial because it creates quantum states encoding higher-order interaction terms that are difficult to represent in shallow classical models. Thus, the ZZFeatureMap embeds the data into a quantum feature space that captures both, linear feature effects (via single-qubit rotations) and second-order feature interactions (via entangling *ZZ* terms). The combination of single-qubit and entangling interactions enables $U_{\textrm{ZZ}}(x)$ to generate a rich family of quantum states whose geometry reflects the input structure. This is particularly advantageous for regression tasks in materials science, where the target quantities (such as optical band gaps) often depend on complex, nonlinear relationships between material descriptors.

### Variational Ansatz

After the classical data have been encoded into quantum states via the feature map, the resulting state $U_{\textrm{ZZ}}(x)|0\rangle$ is processed by a variational quantum circuit (VQC). The VQC implements a family of trainable unitary transformations parameterized by a set of real-valued parameters $\theta = (\theta _1, \theta _2, \ldots , \theta _m)$:

7 $$\begin{aligned} |\psi (x;\theta )\rangle = U(\theta )\, U_{\textrm{ZZ}}(x)\, |0\rangle . \end{aligned}$$

The role of the variational circuit is to learn an optimal transformation of the data-embedded quantum state such that the final measurement statistics approximate the target regression function. Practically, the ansatz is constructed from alternating layers of parameterized single-qubit rotations and multi-qubit entangling gates. The most common parameterized rotations take the form:

8 $$\begin{aligned} R_Y(\theta _k) = e^{-i \theta _k Y/2}, \qquad R_Z(\theta _k) = e^{-i \theta _k Z/2}, \end{aligned}$$

where *Y* and *Z* denote the corresponding Pauli operators. These rotations modulate the amplitudes and phases of individual qubits and introduce trainable degrees of freedom analogous to weights in classical neural networks. Following the local rotations, entangling gates (such as controlled-NOT or controlled-*Z* operations) are applied to couple the qubits. These entangling operations are essential for generating non-classical correlations that allow the quantum circuit to explore a significantly larger portion of the Hilbert space than is accessible through single-qubit operations alone. For an ansatz constructed with *L* repeated layers, the full unitary is expressed as

9 $$\begin{aligned} U(\theta ) = \prod _{l=1}^{L} U_{\text {layer}}(\theta ^{(l)}), \end{aligned}$$

where each layer $U_{\text {layer}}$ contains both parameterized single-qubit rotations and a fixed entangling pattern.

Increasing the number of layers *L* enhances the expressive power of the model, analogous to increasing the depth of a classical neural network. In particular, repeated application of trainable unitaries allows the VQC to approximate highly non-linear functions of the input features. The entanglement between qubits serves as a key resource: it enables the circuit to represent complex joint feature interactions that are difficult to capture using classical models without significant computational overhead. Consequently, variational quantum circuits provide a flexible and highly expressive hypothesis class, capable of approximating a wide range of regression functions when trained with appropriate optimization strategies.

### Quantum measurement, regression output and training procedure

Once the data have been embedded using the feature map and transformed by the variational circuit, the resulting quantum state $|\psi (x;\theta )\rangle$ is measured to produce a regression prediction. In quantum machine learning, the prediction is obtained as the expectation value of a chosen observable *M*, typically a single Pauli operator (such as $Z_k$) or a weighted sum of Pauli operators acting on one or more qubits:

10 $$\begin{aligned} \hat{y}(x;\theta ) = \langle \psi (x;\theta ) |\, M \,| \psi (x;\theta ) \rangle . \end{aligned}$$

Because quantum measurements yield binary outcomes in each execution (or “shot”), the empirical estimate of the expectation value is computed from repeated measurements:

11 $$\begin{aligned} \hat{y}_{\textrm{empirical}} = \frac{1}{S} \sum _{s=1}^{S} m_s, \end{aligned}$$

where $m_s \in \{-1, +1\}$ denotes the outcome obtained during the *s*-th execution and *S* is the total number of measurement shots. Increasing *S* reduces statistical fluctuations and yields a more accurate approximation of the true expectation value.

To train the variational quantum circuit, the parameters *θ* must be iteratively updated using either gradient-based or gradient-free optimization procedures. For variational circuits constructed from parameterized quantum gates of the form

12 $$\begin{aligned} e^{-i\theta P/2}, \end{aligned}$$

where *P* is a Pauli operator, the derivative of any expectation value with respect to the variational parameter can be evaluated exactly using the parameter-shift rule^10, 11^. This rule provides a closed-form expression for the gradient:

13 $$\begin{aligned} \frac{\partial f}{\partial \theta } = \frac{1}{2} \left[ f\!\left( \theta + \frac{\pi }{2}\right) - f\!\left( \theta - \frac{\pi }{2}\right) \right] , \end{aligned}$$

which holds whenever the generator of the gate has only two distinct eigenvalues, a condition met by all single-qubit Pauli rotations used in standard variational ansätze. Because this expression requires only two additional circuit evaluations and does not rely on numerical finite-difference approximations, it yields an unbiased and hardware-compatible estimator of the gradient. For this reason, the parameter-shift rule has become a standard tool for training variational quantum circuits on both simulators and real quantum devices^12^.

For regression problems, such as predicting optical band gaps, the model parameters are optimized by minimizing a cost function that quantifies the discrepancy between predicted and true target values. The standard choice is the mean squared error (MSE):

14 $$\begin{aligned} L(\theta ) = \frac{1}{N} \sum _{i=1}^{N} \left( y_i - \hat{y}(x_i;\theta ) \right) ^2, \end{aligned}$$

where $\{(x_i, y_i)\}_{i=1}^{N}$ is the training dataset. The parameters are iteratively updated according to

15 $$\begin{aligned} \theta _{t+1} = \theta _t - \eta \, \nabla _\theta L(\theta _t), \end{aligned}$$

with learning rate *η*. In practice, both gradient-based optimizers (e.g., Adam) and gradient-free methods (e.g., COBYLA or SPSA) are commonly used depending on the circuit structure, shot noise and hardware constraints. Combining embedding, variational transformation, measurement and optimization yields the complete quantum regression model:

16 $$\begin{aligned} \hat{y}(x;\theta ) = \langle 0 | U_{\textrm{ZZ}}^{\dagger }(x)\, U^{\dagger }(\theta )\, M\, U(\theta )\, U_{\textrm{ZZ}}(x) |0\rangle . \end{aligned}$$

This formulation enables the quantum model to represent nonlinear mappings between input features and continuous outputs. Through both entanglement and variational parameterization, the model can capture higher-order correlations that may be difficult for classical regressors to learn efficiently, making it well-suited for complex tasks in materials science and other data-driven scientific domains.

## Results and discussion

### Experimental results

#### Compositional analysis

**Fig. 1:**
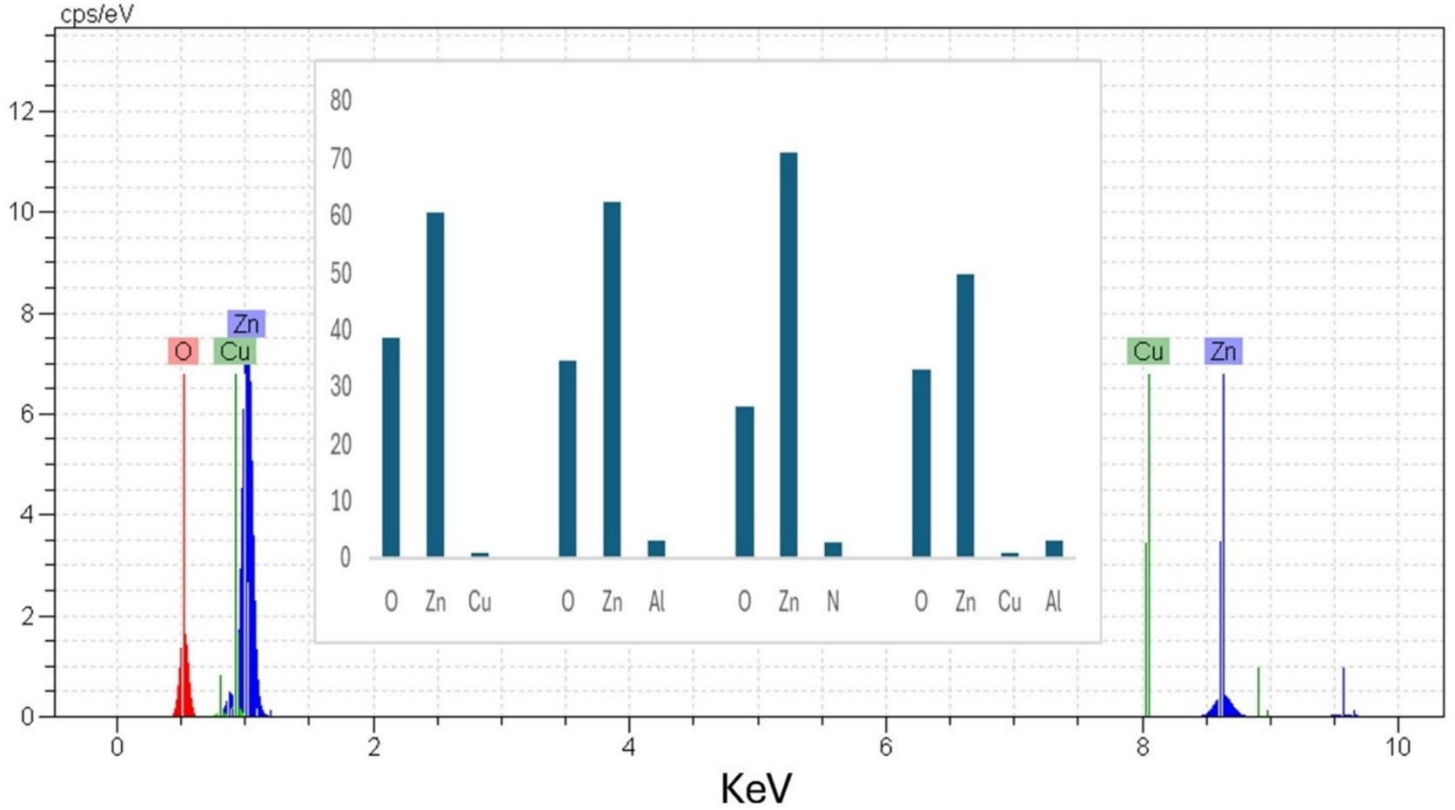
Representative EDS spectrum of a Cu:ZnO thin film. Inset: weight percentages of Zn, O and dopant elements (Cu, N, Al) as determined from EDS analysis.

Semi-quantitative elemental analysis of the deposited films was performed using energy-dispersive X-ray spectroscopy (EDS). This technique was employed to verify dopant incorporation and to assess compositional uniformity. Figure 1 presents a representative EDS spectrum acquired from a Cu-doped ZnO thin film, with the corresponding weight percentages of constituent elements shown in the inset. The spectra confirm the presence of Zn, O, and the respective dopant species (Al, Cu, N) in each film variant. While the Zn and O concentrations remained relatively constant across all samples, minor variations in dopant weight percentages were observed, reflecting differences in sputtering yield and incorporation efficiency among the various target compositions.

#### Structural characterization

**Fig. 2:**
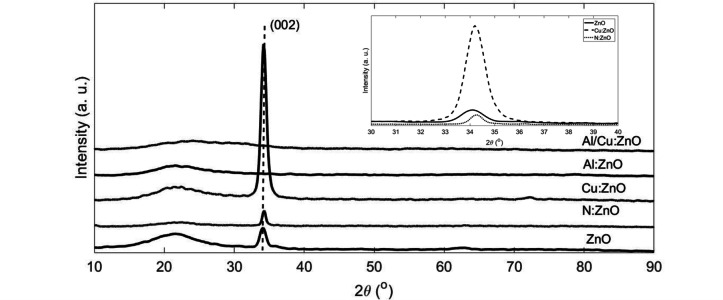
XRD patterns of undoped ZnO and doped/co-doped ZnO thin films deposited on quartz substrates.

X-ray diffraction was employed to investigate the crystallographic structure, phase purity, and preferential orientation of the deposited films. Figure 2 presents the XRD patterns of undoped ZnO and variously doped ZnO thin films. The N:ZnO and Cu:ZnO films exhibit well-defined diffraction peaks consistent with the hexagonal wurtzite structure of ZnO (JCPDS card no. 01-079-0207). A prominent (002) reflection is observed in all crystalline samples, indicating strong preferential orientation along the *c*-axis perpendicular to the substrate surface, a characteristic feature of sputtered ZnO films grown under optimized conditions.

In contrast, the Al:ZnO and Al/Cu:ZnO co-doped films deposited without post-annealing exhibit no discernible diffraction peaks, indicating an amorphous microstructure. This amorphization is attributed to the high concentration of Al dopants, which disrupt the long-range crystallographic order during film growth by introducing substantial lattice strain and inhibiting grain nucleation.

Systematic shifts in the (002) peak position were observed as a function of dopant species. The Cu:ZnO films exhibited a shift toward higher *2θ* angles relative to undoped ZnO, consistent with compressive lattice strain arising from the substitution of Zn^2+^ ions (ionic radius: 0.74 Å) by the slightly smaller Cu^2+^ ions (ionic radius: 0.73 Å)^13^

**Fig. 3:**
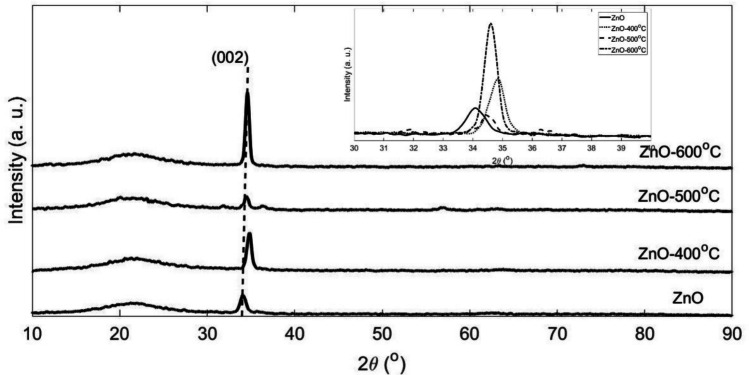
XRD patterns of undoped ZnO thin films: as-deposited and annealed at 400, 500 and 600 °C.

The influence of post-deposition annealing on the crystallographic properties of undoped ZnO films is illustrated in Fig.  3. The as-deposited film exhibits a single (002) reflection at *2θ = 34.07*°, confirming the wurtzite phase with *c*-axis orientation. Upon annealing at 400 and 600 °C, the (002) peak shifts to *2θ = 34.89*° and 34.59°, respectively, accompanied by progressive peak narrowing indicative of crystallite growth and strain relaxation.

Notably, the film annealed at 500 °C exhibits a markedly different diffraction pattern, with four distinct reflections corresponding to the (100), (002), (101), and (110) planes observed at *2θ = 31.85*°, 34.46°, 36.35°, and 56.75°, respectively. This emergence of multiple crystallographic orientations suggests that 500 °C represents a critical transition temperature at which significant microstructural reorganization occurs, possibly driven by the release of trapped oxygen from vacancy sites and consequent stoichiometry modification. Similar phenomena have been attributed to enhanced atomic mobility at elevated temperatures, which promotes grain boundary migration and crystallite coalescence^14^.

**Fig. 4:**
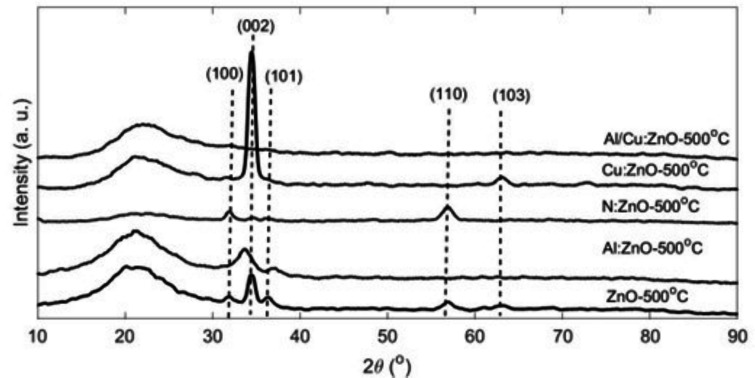
XRD patterns of undoped ZnO and doped/co-doped ZnO thin films annealed at 500 °C.

The annealing-induced crystallization of initially amorphous films is demonstrated in Fig.  4, which compares the XRD patterns of all film variants annealed at 500 °C. The Al:ZnO film, which was amorphous in the as-deposited state, transforms into a crystalline phase upon annealing, exhibiting a reflection near *2θ = 36.89*°. This thermally activated crystallization indicates that sufficient atomic mobility is achieved at 500 °C to overcome the kinetic barriers imposed by Al-induced lattice distortion. The N:ZnO films annealed at 500 °C display additional (100) and (101) reflections, while the Cu:ZnO films exhibit an emerging (103) plane alongside the dominant (002) reflection, suggesting dopant-dependent variations in the preferred crystallographic orientation.

The interplanar spacing ($d_{hkl}$) for the hexagonal wurtzite structure was calculated using the standard relation:

17 $$\begin{aligned} \frac{1}{d_{hkl}^2} = \frac{4}{3}\left( \frac{h^2 + hk + k^2}{a^2}\right) + \frac{l^2}{c^2} \end{aligned}$$

where *a* and *c* are the lattice parameters, and (*h*, *k*, *l*) are the Miller indices of the diffracting plane.

The average crystallite size (*D*) was estimated from the full width at half maximum (FWHM) of the (002) reflection using the Scherrer equation^15^:

18 $$\begin{aligned} D = \frac{K\lambda }{\beta \cos \theta } \end{aligned}$$

where *K = 0.9* is the shape factor, *λ = 0.15406* nm is the Cu-K*α* wavelength, *β* is the FWHM in radians, and *θ* is the Bragg angle. The lattice parameter *c* was determined from the (002) reflection using^16^:

19 $$\begin{aligned} c = \frac{\lambda }{\sin \theta } \end{aligned}$$

Table 2 summarizes the structural parameters extracted from XRD analysis for all film variants. This approach provides an approximate measure of the coherent diffraction domain size. As instrumental broadening was not explicitly corrected, the value obtained represents lower-bound estimates. Furthermore, the observed peak broadening may arise from combined contributions of finite crystallite size, microstrain, defects, and structural disorder-particularly in doped and partially amorphous samples. Accordingly, the calculated crystallite sizes are used primarily for comparative analysis among samples rather than as absolute values. Several trends are evident from these data. First, annealing generally promotes crystallite growth: the crystallite size of undoped ZnO increases from 25.17 nm (as-deposited) to 37.25 nm after annealing at 600 °C, accompanied by a reduction in FWHM from 0.576° to 0.223°. Second, the lattice parameter *c* decreases with increasing annealing temperature (from 5.26 Å to 5.18 Å for undoped ZnO), reflecting strain relaxation toward the bulk ZnO value ($c_0 = 5.206$ Å). Third, dopant incorporation induces distinct lattice distortions: Cu:ZnO films exhibit slightly reduced *c* values due to the smaller ionic radius of Cu^2+^, while Al:ZnO films show variable lattice parameters depending on the degree of crystallization.

**Table 2:** Structural parameters of undoped and doped ZnO thin films extracted from XRD analysis.

Sample	*2θ* (deg)	*c* (Å)	FWHM (deg)	*D* (nm)
ZnO (as-deposited)	34.07	5.26	0.576	25.2
ZnO (400 °C)	34.89	5.14	0.528	27.6
ZnO (500 °C)	34.46	5.20	0.394	36.9
ZnO (600 °C)	34.59	5.18	0.223	37.3
N:ZnO (300 °C)	34.27	5.23	0.336	24.7
N:ZnO (400 °C)	34.51	5.19	0.472	17.6
N:ZnO (500 °C)	34.67	5.19	0.020	41.6
N:ZnO (600 °C)	34.57	5.18	0.629	13.2
Al:ZnO (400 °C)	36.69	4.90	1.065	13.7
Al:ZnO (500 °C)	36.89	5.29	0.821	18.2
Cu:ZnO (as-deposited)	34.20	5.24	0.787	10.6
Cu:ZnO (500 °C)	34.37	5.20	0.336	24.7

The XRD analysis thus provides a comprehensive picture of the structural evolution of ZnO thin films as a function of both dopant incorporation and thermal treatment. Annealing promotes grain growth, strain relaxation, and improved crystallinity, as evidenced by peak sharpening and shifts toward bulk lattice parameter values. Dopant substitution introduces characteristic lattice distortions that depend on the ionic radius mismatch between the dopant and host Zn^2+^ ions. These structural modifications directly influence the optical and electronic properties of the films, establishing a clear correlation between processing conditions and material functionality.

#### Surface morphology

**Fig. 5:**
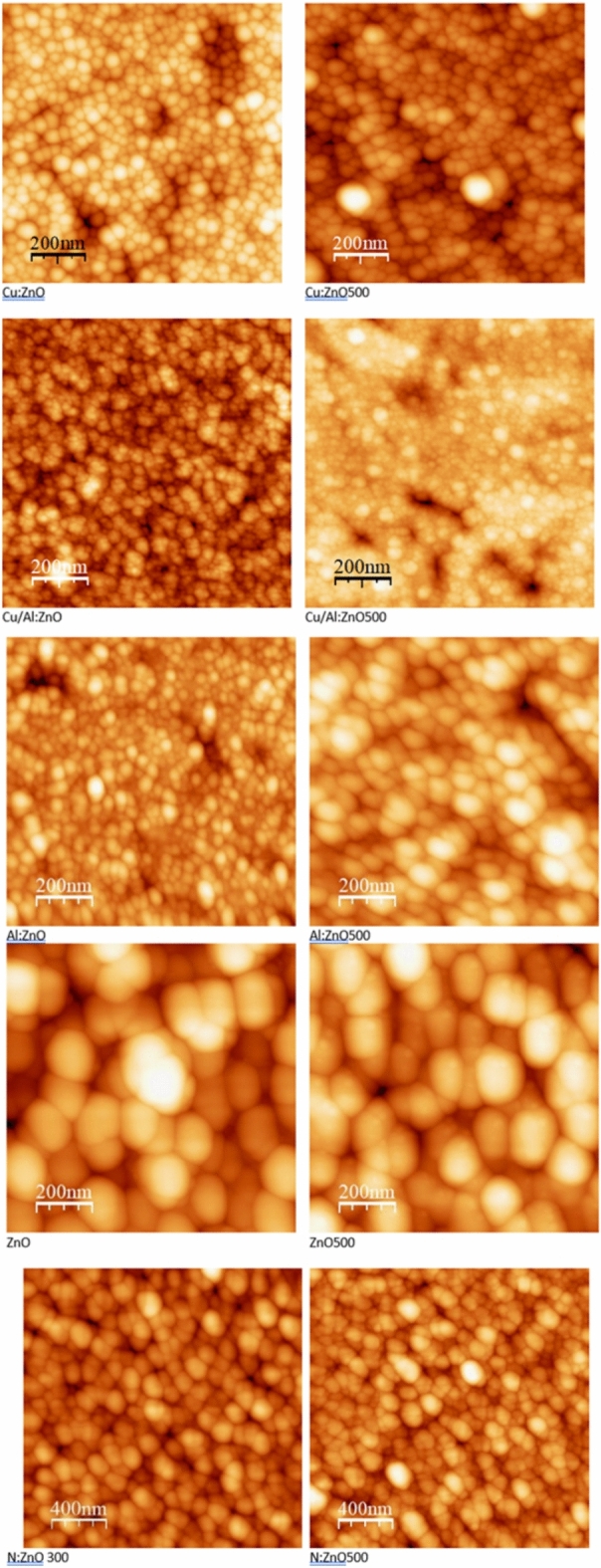
AFM images of ZnO and doped ZnO thin films.

Atomic force microscopy was employed to characterize the surface topography and grain structure of the deposited films. Figure 5 presents representative AFM images acquired over a $2~\mu \text {m} \times 2~\mu \text {m}$ scan area for selected film variants, both as-deposited and after annealing at 500 °C. The root-mean-square (RMS) surface roughness values extracted from these measurements are summarized in Table 3.

The AFM analysis reveals dopant-specific effects on surface morphology that correlate with the structural observations from XRD. All films exhibit RMS roughness values in the range of 0.94–9.67 nm, which is significantly smaller than the film thicknesses (230–300 nm), confirming the formation of smooth, continuous films suitable for device applications.

The influence of annealing temperature on surface morphology follows a non-monotonic trend. For N:ZnO films, the RMS roughness decreases progressively from 9.67 nm at 300 °C to 4.90 nm at 600 °C, indicating grain coalescence and surface smoothening driven by thermally activated atomic diffusion. In contrast, undoped ZnO films exhibit an initial increase in roughness upon annealing at 400 °C (from 5.94 to 8.21 nm), followed by a decrease at 500 °C (6.23 nm), reflecting competitive processes of grain growth and surface reorganization.

Each dopant species induces characteristic morphological signatures. Aluminum doping produces the smoothest surfaces (RMS = 2.58–4.06 nm), consistent with the inhibition of grain growth observed in the XRD analysis and the formation of fine-grained or partially amorphous microstructures. Copper-doped films exhibit moderate roughness values (3.57–7.98 nm) and relatively uniform grain distributions, suggesting that Cu promotes stable grain growth without excessive surface irregularities. Nitrogen incorporation tends to increase surface roughness, particularly at lower annealing temperatures, which is attributed to defect formation and lattice distortion associated with N_O_ substitution and compensating defect complexes.

The combined AFM and XRD analysis demonstrates that the interplay between dopant chemistry and thermal treatment governs the evolution of both bulk crystallinity and surface morphology. Optimal surface quality is typically achieved at intermediate annealing temperatures of 400–500 °C. In this range, sufficient atomic mobility promotes grain coalescence and produces low roughness with a uniform grain structure. At the same time, excessive grain coarsening or dopant diffusion is avoided, preserving surface homogeneity.

#### Optical properties

**Fig. 6:**
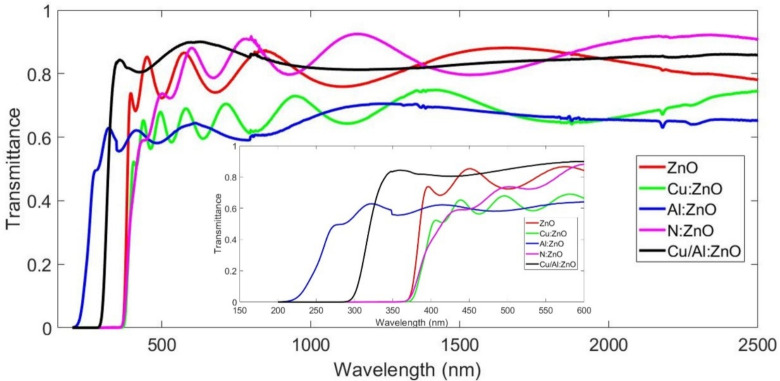
Optical transmittance spectra of undoped ZnO and doped/co-doped ZnO thin films deposited on quartz substrates.

The optical transmittance spectra of undoped and doped ZnO thin films, measured over the wavelength range of 200–2500 nm, are presented in Fig.  6. All films exhibit high optical transparency in the visible region (400-700 nm), with average transmittance values exceeding 60-90%, confirming their suitability for transparent conductive oxide applications. The absorption edge, corresponding to the onset of fundamental band-to-band transitions, is clearly visible in the near-ultraviolet region and exhibits systematic shifts depending on dopant species and annealing conditions.

The absorption coefficient (*α*) was calculated from the transmittance data using the relation:

20 $$\begin{aligned} \alpha = \frac{1}{d}\ln \left( \frac{1}{T}\right) \end{aligned}$$

where *d* is the film thickness and *T* is the optical transmittance.

**Fig. 7:**
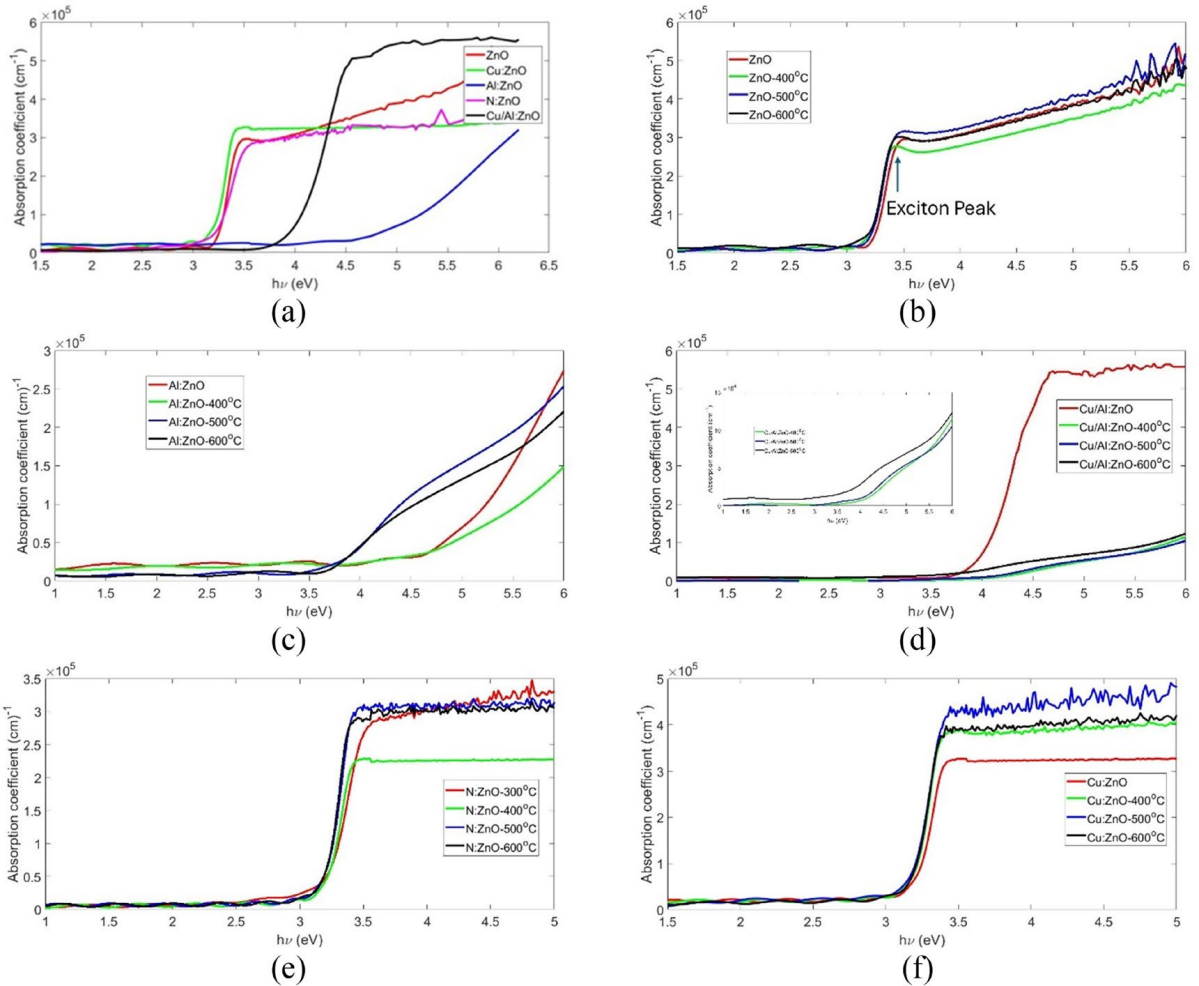
Absorption coefficient versus photon energy for (**a**) undoped ZnO and selected doped/co-doped ZnO thin films, (**b**) undoped ZnO thin films annealed at 400, 500, and 600 °C, (**c**) Al:ZnO thin films annealed at 400, 500, and 600 °C, (**d**) Cu/Al:ZnO thin films annealed at 400, 500, and 600 °C, (**e**) N:ZnO thin films annealed at 300, 400, 500, and 600 °C, (**f**) Cu:ZnO thin films annealed at 400, 500, and 600 °C.

**Fig. 8:**
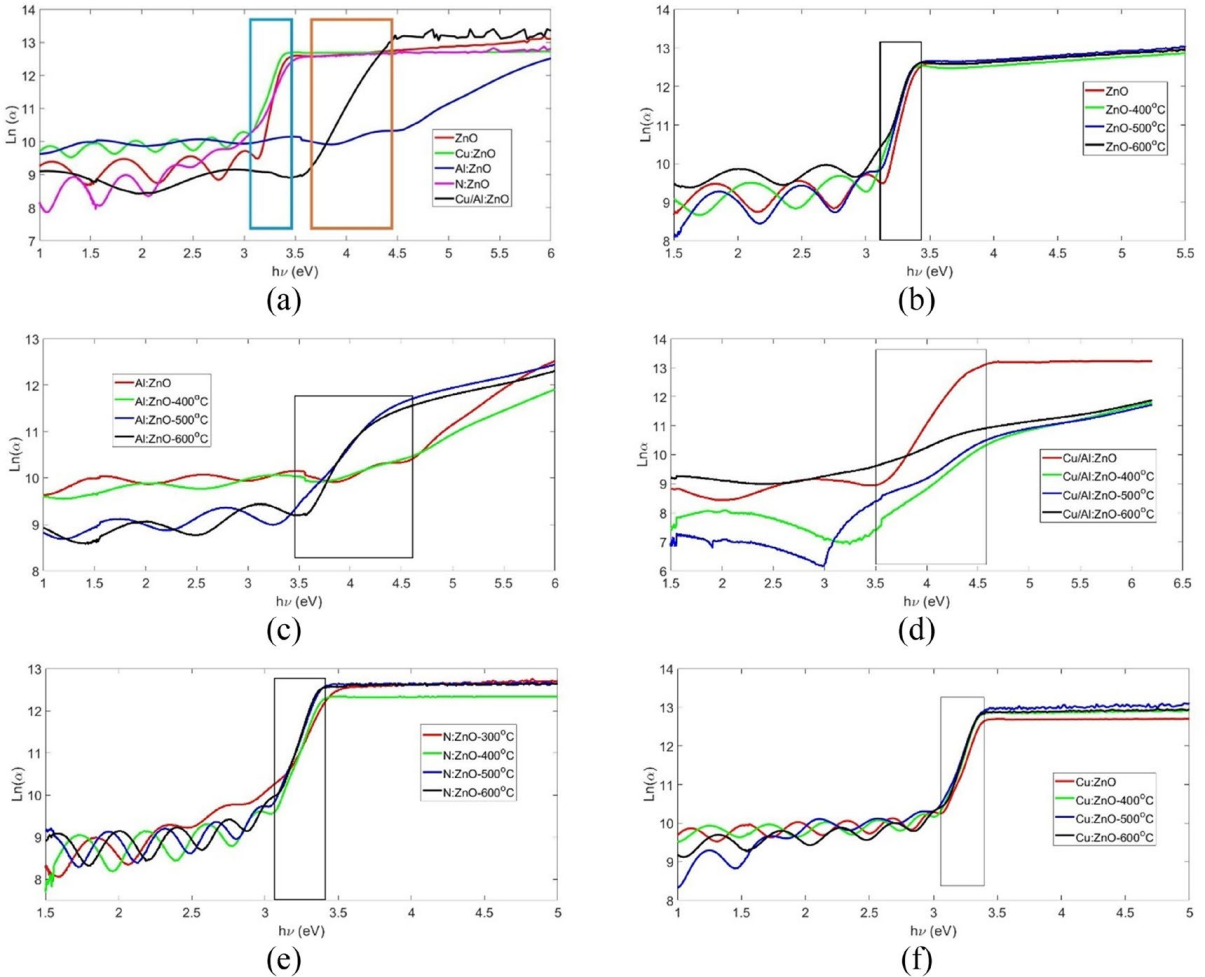
Plot of $\ln (\alpha )$ versus photon energy for (**a**) undoped ZnO and doped/co-doped ZnO thin films and the shaded regions (orange and blue) highlight the Urbach tail regimes used for linear fitting, (**b**) undoped ZnO thin films annealed at 400, 500, and 600 °C, (**c**) Al:ZnO thin films annealed at 400, 500, and 600 °C, (**d**) Cu/Al:ZnO thin films annealed at 400, 500, and 600 °C, (**e**) N:ZnO thin films annealed at 300, 400, 500, and 600 °C, (**f**) Cu:ZnO thin films annealed at 400, 500, and 600 °C.

Figure 7 presents the absorption coefficient as a function of photon energy for selected doped and co-doped ZnO films, while Fig. 7b shows the corresponding spectra for undoped ZnO films annealed at different temperatures. The absorption edge shifts systematically with both dopant incorporation and annealing temperature, reflecting modifications to the electronic band structure (Fig.  8).

The optical band gap of ZnO and doped ZnO thin films deposited on quartz substrates is calculated using the following Eq. (21)^17^:

21 $$\begin{aligned} {(\alpha h\nu )^{1/m}} = c(h\nu - {E_g}) \end{aligned}$$

**Fig. 9:**
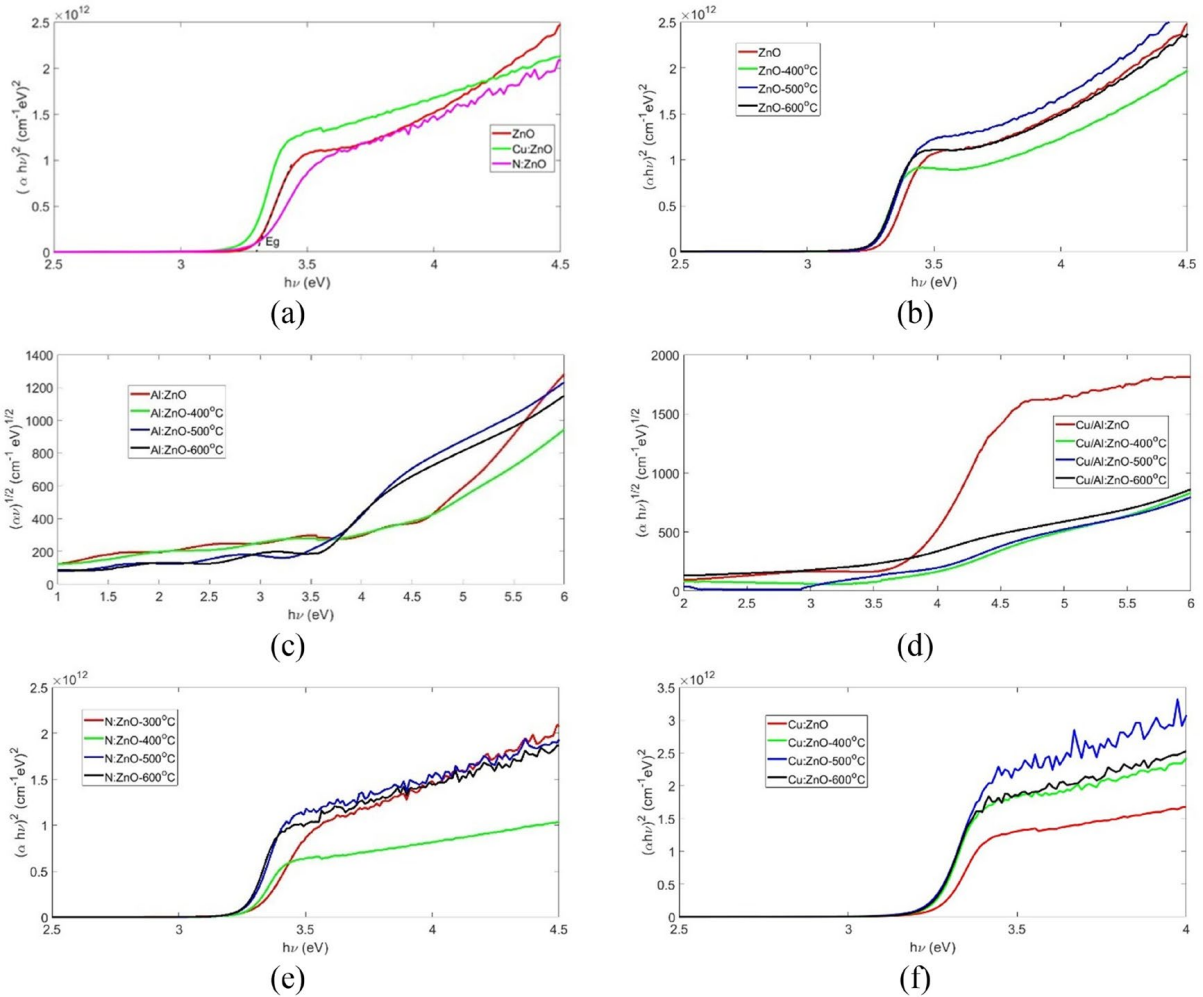
Tauc plots ($(\alpha h\nu )^2$ versus *hν*) for (**a**) undoped ZnO and selected doped/co-doped ZnO thin films, (**b**) undoped ZnO thin films annealed at 400, 500, and 600 °C, (**e**) N:ZnO thin films annealed at 300, 400, 500, and 600 °C, (**f**) Cu:ZnO thin films annealed at 400, 500, and 600 °C, Tauc plots ($(\alpha h\nu )^{1/2}$ versus *hν*) for (**c**) Al:ZnO thin films annealed at 400, 500, and 600 °C, (**d**) Cu/Al:ZnO thin films annealed at 400, 500, and 600 °C.

The bandgap was extracted by linear extrapolation of the $(\alpha h\nu )^2$ versus *hν* plot to the energy axis intercept ($(\alpha h\nu )^2 = 0$) (Fig. 9). The extracted bandgap values are summarized in Table 3.

The Urbach energy ($E_\textrm{U}$), which quantifies the width of exponential band-tail states arising from structural disorder, defects, and compositional fluctuations, was determined from the sub-bandgap absorption region using:

22 $$\begin{aligned} \alpha = \alpha _0 \exp \left( \frac{h\nu }{E_\textrm{U}}\right) \end{aligned}$$

where $\alpha _0$ is a pre-exponential constant. The Urbach energy was extracted from the inverse slope of linear fits to $\ln (\alpha )$ versus *hν* plots in the exponential tail region (Fig.  8).

**Fig. 10:**
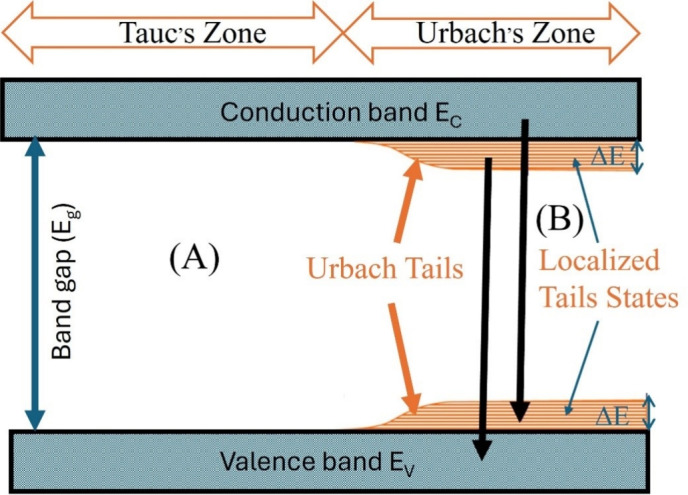
Schematic diagram of the density of states and energy. (**A**) Tauc’s Zone, (**B**) Urbach’s Zone.

The Urbach energy quantifies static, structural disorder causing localized exponential-tail states, and dynamic disorder from electron-phonon scattering. According to Fig. 10, the value of *Δ E* is the electronic width near the edges of the conduction band and the valence band, which depends on the Urbach energy. The value of *Δ E* in the band gap can be calculated by Urbach energy. Urbach energy ($E_u$) is the width of the localized states and is defined in semiconductors where the absorption in the band gap is broadened due to various defects in the crystal structure. The ratio of the number of defect and weak bonds to that of the extended states should influence the ratio of their widths. Also, the ratio of the effective mass of charge carriers in the tail states ($E_u$) to those in extended states depends on the ratio of width of the tail states to that of extended states. Hence the position of $E_g$ should depend on such ratio.

The Urbach energy values, presented in Table 3, reveal significant variations with dopant type. Undoped ZnO exhibits the lowest Urbach energy (74.9 meV), indicating minimal structural disorder. Nitrogen and copper doping produce modest increases in $E_\textrm{U}$ (96–150 meV), reflecting the introduction of point defects and shallow localized states. In contrast, aluminum doping and Al/Cu co-doping result in substantially elevated Urbach energies (200–978 meV), consistent with the amorphous or highly disordered microstructures observed by XRD for these compositions. The broad Urbach tails in Al-containing films indicate extensive band-tail disorder arising from the high dopant concentration and associated lattice strain.

The optical properties of the ZnO thin films exhibit systematic dependences on both dopant species and annealing temperature, as summarized in Table 3 and illustrated schematically in the following analysis.

*Undoped ZnO:* The as-deposited film exhibits a bandgap of 3.30 eV, which decreases slightly to 3.26 eV upon annealing at 400–600 °C. This bandgap narrowing is attributed to crystallite growth and the reduction of quantum confinement effects, consistent with the XRD observation of increasing crystallite size with annealing temperature.

*Aluminum-doped ZnO:* Al:ZnO films display the largest bandgaps, reaching 4.0 eV in the as-deposited (amorphous) state. Upon annealing, the bandgap decreases progressively to 3.30–3.60 eV as the films crystallize. The bandgap widening relative to undoped ZnO is explained by the Burstein-Moss effect^14^: Al^3+^ substitution for Zn^2+^ introduces additional electrons that populate the conduction band, raising the Fermi level and increasing the optical transition energy. Concurrently, the Urbach energy decreases with annealing as structural disorder is reduced.

*Copper-doped ZnO:* Cu:ZnO films exhibit bandgaps slightly lower than undoped ZnO (3.25–3.28 eV), with minimal variation upon annealing. Copper introduces localized *d*-states near the valence band maximum, which act as acceptor-like levels and effectively narrow the optical bandgap. The relatively constant Urbach energy (99–141 meV) indicates moderate structural disorder that is insensitive to thermal treatment within the investigated temperature range.

*Nitrogen-doped ZnO:* N:ZnO films maintain bandgaps close to undoped ZnO (3.26–3.30 eV) across all annealing temperatures, suggesting that nitrogen incorporation at the levels achieved does not significantly alter the fundamental band structure. However, the elevated Urbach energies (96–150 meV) indicate increased sub-bandgap absorption associated with nitrogen-related defect states and compensating complexes (e.g., N_O_–V_O_).

*Al/Cu co-doped ZnO:* The co-doped Al/Cu films exhibit complex optical behavior, with bandgaps ranging from 2.6 to 3.8 eV depending on annealing conditions. The as-deposited films show bandgap widening (3.75-3.80 eV), which can be attributed to Al-induced Burstein-Moss effects. Upon annealing at 600 °C, a pronounced bandgap reduction to  2.6 eV is observed. This anomalous narrowing may be associated with several possible factors, including dopant redistribution, defect-induced band tailing, or the formation of secondary phases (e.g., CuO or Cu_2_O); however, these mechanisms cannot be conclusively confirmed based on the present data and should be regarded as plausible hypotheses. The corresponding increase in Urbach energy (978 meV at 600  °C) suggests enhanced structural disorder, which may contribute to the observed bandgap narrowing. Although the large Urbach energy values mainly indicate significant intrinsic disorder and defect-induced band tailing within the ZnO lattice, additional contributions from optical scattering, porous morphology, and thickness nonuniformity cannot be completely excluded. Surface roughness and microstructural inhomogeneity may broaden the absorption edge and partially increase the extracted Urbach energy values. Therefore, the reported Urbach energies likely reflect a combined influence of intrinsic electronic disorder and extrinsic optical effects.

**Table 3:** Optical and morphological parameters of undoped and doped ZnO thin films.

Sample	$E_\textrm{U}$ (eV)	RMS roughness (nm)	$E_\textrm{g}$ (eV)
ZnO (as-deposited)	0.074	5.94	3.30
ZnO (400 °C)	0.092	8.21	3.26
ZnO (500 °C)	0.080	6.23	3.28
ZnO (600 °C)	0.094	7.39	3.26
N:ZnO (300 °C)	0.150	9.67	3.30
N:ZnO (400 °C)	0.112	6.39	3.28
N:ZnO (500 °C)	0.109	6.26	3.27
N:ZnO (600 °C)	0.096	4.90	3.26
Al:ZnO (as-deposited)	0.705	3.50	4.00
Al:ZnO (400 °C)	0.942	4.06	3.60
Al:ZnO (500 °C)	0.396	2.58	3.30
Al:ZnO (600 °C)	0.259	3.87	3.35
Cu:ZnO (as-deposited)	0.119	4.10	3.28
Cu:ZnO (400 °C)	0.113	4.33	3.26
Cu:ZnO (500 °C)	0.141	3.57	3.25
Cu:ZnO (600 °C)	0.099	7.98	3.26
Al/Cu:ZnO (as-deposited)	0.200	0.94	3.75
Al/Cu:ZnO (400 °C)	0.396	3.37	3.50
Al/Cu:ZnO (500 °C)	0.509	5.49	3.40
Al/Cu:ZnO (600 °C)	0.978	5.39	2.60

The optical properties of the ZnO thin films are intimately connected to their structural characteristics. The inverse relationship between crystallite size and bandgap observed for undoped ZnO films upon annealing is consistent with quantum confinement theory: as crystallite size increases beyond the exciton Bohr radius (*∼*2 nm for ZnO), confinement effects diminish and the bandgap approaches the bulk value. Simultaneously, improved crystallinity reduces the density of defect states, leading to decreased Urbach energy and sharper absorption edges^18^.

The dopant-induced modifications follow distinct mechanisms. Aluminum introduces shallow donor states and elevates the Fermi level, producing Burstein-Moss bandgap widening. Copper creates deep acceptor-like states that narrow the effective bandgap through transitions involving localized *d*-levels. Nitrogen incorporation generates compensating defect complexes that increase sub-bandgap absorption without substantially shifting the fundamental absorption edge. The co-doped Al/Cu system exhibits competition between these mechanisms, with the dominant effect depending on the relative dopant concentrations and their distribution within the lattice.

The enhancement of optical path length in microcrystalline and nanocrystalline ZnO films can be attributed to microstructure-induced light scattering and photo-trapping effects. Grain boundaries, pores, surface roughness, and nanocrystalline domains create local refractive-index fluctuations that act as optical scattering centers. As a result, incident photons are redirected into oblique and non-linear propagation paths rather than passing directly through the film, leading to multiple internal reflections and an increase in the effective optical path length beyond the physical thickness of the layer.

Furthermore, the broad grain-size distribution obtained from XRD analysis, and the porous morphology observed by AFM suggest the presence of numerous scattering centers and dielectric discontinuities between ZnO and air regions. These structural features further strengthen diffuse scattering and internal reflection effects, contributing to improved light trapping and enhanced optical penetration in the doped ZnO thin films.

These structure-property correlations provide the foundation for the machine-learning analysis presented in the following section, where the relationships between processing parameters, structural descriptors, and optical bandgap are quantitatively modeled to enable predictive optimization of ZnO thin film properties.

### Quantum machine learning results

To investigate the relationship between structural, morphological and optical properties of ZnO-based thin films, four different datasets (Sample Sets 1–4) were prepared. Each dataset contains experimentally measured features describing microstructural quality, impurity incorporation and surface morphology. These datasets enable a systematic analysis of how selected physical parameters influence the optical band gap ($E_g$).

#### Description of the four sample sets

Sample Set 1 comprises Urbach energy (EU), RMS surface roughness and film thickness, representing the combined influence of *electronic disorder*, *surface morphology* and *geometrical confinement* on the optical band gap of ZnO-based thin films (Table 4). Sample Set 2 includes RMS roughness, lattice parameter *c* and crystallite size, providing insight into the effects of *lattice strain*, *grain growth* and *microstructural variations* on optical transitions (Table 5). Sample Set 3 consists of Urbach energy, RMS roughness and lattice parameter *c*, emphasizing the interplay between *electronic disorder* and *crystal lattice distortion* in determining the optical band gap (Table 6). Sample Set 4 contains Urbach energy, RMS roughness and crystallite size, highlighting the roles of *defects*, *grain-boundary scattering* and *microstructural evolution* in tuning the band gap (Table 7).

**Table 4:** Sample Set 1: Urbach energy, RMS roughness, thickness, and optical bandgap of ZnO-based thin films.

Sample	$E_\textrm{U}$ (eV)	RMS (nm)	Thickness (nm)	$E_\textrm{g}$ (eV)
ZnO (as-deposited)	0.075	5.94	230	3.30
ZnO (400 °C)	0.093	8.21	230	3.26
ZnO (500 °C)	0.081	6.23	230	3.28
ZnO (600 °C)	0.095	7.39	230	3.26
N:ZnO (300 °C)	0.150	9.67	300	3.30
N:ZnO (400 °C)	0.112	6.39	300	3.28
N:ZnO (500 °C)	0.109	6.26	300	3.27
N:ZnO (600 °C)	0.096	4.90	300	3.26
Al:ZnO (400 °C)	0.942	4.06	230	3.60
Al:ZnO (500 °C)	0.396	2.58	230	3.30
Al:ZnO (600 °C)	0.259	3.87	230	3.35
Cu:ZnO (as-deposited)	0.119	4.10	230	3.28
Cu:ZnO (400 °C)	0.113	4.33	230	3.26
Cu:ZnO (500 °C)	0.141	3.57	230	3.25
Cu:ZnO (600 °C)	0.099	7.98	230	3.26
Al/Cu:ZnO (as-deposited, 230 nm)	0.200	0.94	230	3.75
Al/Cu:ZnO (400 °C)	0.396	3.37	230	3.50
Al/Cu:ZnO (500 °C)	0.509	5.49	230	3.40
Al/Cu:ZnO (as-deposited, 50 nm)	0.490	5.56	50	3.80
Al/Cu:ZnO (as-deposited, 150 nm)	0.230	3.13	150	3.70

**Table 5:** Sample Set 2: RMS roughness, lattice parameter *c*, crystallite size, and optical bandgap of ZnO-based thin films.

Sample	RMS (nm)	*c* (Å)	*D* (nm)	$E_\textrm{g}$ (eV)
ZnO (as-deposited)	5.94	5.26	25.2	3.30
ZnO (400 °C)	8.21	5.14	27.6	3.26
ZnO (500 °C)	6.23	5.20	36.9	3.28
ZnO (600 °C)	7.39	5.18	37.3	3.26
N:ZnO (300 °C)	9.67	5.23	24.7	3.30
N:ZnO (400 °C)	6.39	5.19	17.6	3.28
N:ZnO (500 °C)	6.26	5.19	41.6	3.27
N:ZnO (600 °C)	4.90	5.18	13.2	3.26
Al:ZnO (400 °C)	4.06	4.90	13.7	3.60
Al:ZnO (500 °C)	2.58	5.29	18.0	3.30
Cu:ZnO (as-deposited)	4.10	5.24	10.6	3.28
Cu:ZnO (500 °C)	3.57	5.20	24.7	3.25
Al/Cu:ZnO (600 °C)	5.39	5.24	13.5	2.60

**Table 6:** Sample Set 3: Urbach energy, RMS roughness, lattice parameter *c*, and optical bandgap of ZnO-based thin films.

Sample	$E_\textrm{U}$ (eV)	RMS (nm)	*c* (Å)	$E_\textrm{g}$ (eV)
ZnO (as-deposited)	0.075	5.94	5.26	3.30
ZnO (400 °C)	0.093	8.21	5.14	3.26
ZnO (500 °C)	0.081	6.23	5.23	3.28
ZnO (600 °C)	0.095	7.39	5.18	3.26
N:ZnO (300 °C)	0.150	9.67	5.23	3.30
N:ZnO (400 °C)	0.112	6.39	5.19	3.28
N:ZnO (500 °C)	0.109	6.26	5.19	3.27
N:ZnO (600 °C)	0.096	4.90	5.18	3.26
Al:ZnO (500 °C)	0.390	2.58	5.29	3.40
Cu:ZnO (as-deposited)	0.091	3.81	5.24	3.27
Cu:ZnO (500 °C)	0.089	3.23	5.20	3.25
Al/Cu:ZnO (600 °C)	0.978	5.39	5.24	2.60

**Table 7:** Sample Set 4: Urbach energy, RMS roughness, crystallite size, and optical bandgap of ZnO-based thin films.

Sample	$E_\textrm{U}$ (eV)	RMS (nm)	*D* (nm)	$E_\textrm{g}$ (eV)
ZnO (as-deposited)	0.075	5.94	14.4	3.30
ZnO (400 °C)	0.093	8.21	15.8	3.26
ZnO (500 °C)	0.081	6.23	21.1	3.28
ZnO (600 °C)	0.095	7.39	12.4	3.26
N:ZnO (300 °C)	0.150	9.67	24.7	3.30
N:ZnO (400 °C)	0.112	6.39	17.6	3.28
N:ZnO (500 °C)	0.109	6.26	41.6	3.27
N:ZnO (600 °C)	0.096	4.90	13.2	3.26
Al:ZnO (500 °C)	0.396	2.58	18.0	3.30
Al:ZnO (600 °C)	0.259	3.87	39.0	3.35
Cu:ZnO (as-deposited)	0.119	4.10	10.6	3.28
Cu:ZnO (500 °C)	0.141	3.57	24.7	3.25
Al/Cu:ZnO (600 °C)	0.978	5.39	13.5	2.60

#### Quantum machine learning results

This section presents the complete quantum machine learning results obtained using the Variational Quantum Regressor (VQR) for four distinct ZnO-based thin?film datasets. Each sample set contains a different combination of structural, morphological and electronic features used to predict the optical band gap $E_g$. All models were trained using an identical quantum circuit architecture to ensure fair comparison across datasets. The quantum circuit consisted of a ZZFeatureMap with full entanglement and five repetitions, combined with a TwoLocal ansatz (ry/rz rotations and cz entanglement) with five repetitions. The optimizer used was L–BFGS–B with a maximum iteration count of 500. The total number of trainable parameters was 36, with a feature?map depth of 55 and ansatz depth of 27.

The VQR regression model was trained and evaluated separately for each dataset. Table 8 summarizes the overall performance metrics computed from the complete set of predictions across all samples in each dataset.

**Table 8:** Overall performance summary of the VQR model for Sample Sets 1–4.

Metric	Set 1 ($N{=}20$)	Set 2 ($N{=}13$)	Set 3 ($N{=}12$)	Set 4 ($N{=}13$)
Overall $R^{2}$	0.425	0.488	0.541	0.506
Overall MAE (eV)	0.089	0.094	0.058	0.058
Overall RMSE (eV)	0.135	0.148	0.131	0.129

Across the four datasets, the VQR model explains 43-54% of the total variance in optical band gaps, with overall MAE values ranging from 0.058 to 0.094 eV. Sample Set 3, which combines electronic disorder ($E_{\textrm{U}}$), surface morphology (RMS roughness) and lattice strain (*c*-axis parameter), delivered the best overall accuracy (MAE *= 0.058* eV, $R^{2} = 0.541$). Sample Set 4, substituting crystallite size for the lattice parameter, achieved comparable accuracy (MAE *= 0.058* eV, $R^{2} = 0.506$). Sample Sets 1 and 2, which lack the Urbach energy descriptor, yielded higher MAE values (0.089 and 0.094 eV, respectively) and lower $R^{2}$ (0.425 and 0.488), confirming that datasets combining electronic disorder with structural and morphological features provide the most informative input for quantum regression of optical band gaps. A comparative overview of the four datasets reveals several key insights:

- *Best accuracy*: Sample Set 3 (MAE *= 0.058* eV, $R^{2} = 0.541$).
- *Second best*: Sample Set 4 (MAE *= 0.058* eV, $R^{2} = 0.506$).
- *Highest explained variance*: Sample Set 3, with the largest overall $R^{2}$ (0.541).
- *Lowest explained variance*: Sample Set 1 ($R^{2} = 0.425$).

Overall, the prediction errors (0.058–0.094 eV overall MAE) fall within the typical experimental uncertainty in optical band gap extraction ($\pm \,0.05$–0.10 eV), indicating that the VQR model captures the dominant structure-property relationships governing band gap modulation in doped and annealed ZnO thin films. A detailed validation study for Sample Set 3, including cross-validation, classical baseline comparison and experimental uncertainty analysis, is presented in Subsect. “Extended VQR validation and benchmarking for Sample Set 3”.

Tables 9, 10, 11 and 12 provides the complete set of quantum regression outputs for all four datasets used in this work. For each dataset, we report the actual experimentally measured optical band gap values together with the predictions generated by the optimized Variational Quantum Regressor (VQR). To enable precise assessment of the model’s behavior, the tables list absolute prediction errors as well as percentage errors for every individual sample. Presenting the full prediction matrix for each dataset allows a deeper examination of how the quantum model responds to different feature combinations, particularly when the datasets include varying contributions of electronic disorder, surface morphology, crystallite size, lattice strain, or film thickness. These detailed tables highlight not only the overall accuracy metrics but also the specific cases where the quantum model succeeds or struggles, such as samples with outlier structural parameters or extreme Urbach energies. This level of transparency is essential for evaluating quantum machine learning models, as their behavior can depend sensitively on the expressivity of the quantum circuit and the structure of the input features. The following tables therefore provide an essential complement to the global performance metrics reported earlier and allow researchers to inspect the fine-grained predictive performance across all sample sets.

**Table 9:** Detailed quantum machine-learning predictions for Sample Set 1.

Index	Sample	Actual (eV)	Predicted (eV)	Error (eV)	Error (%)
0	ZnO (as-deposited)	3.300	3.313	0.013	0.4
1	ZnO (400 °C)	3.260	3.317	0.057	1.8
2	ZnO (500 °C)	3.280	3.358	0.078	2.4
3	ZnO (600 °C)	3.260	3.358	0.098	3.0
4	N:ZnO (300 °C)	3.300	3.345	0.045	1.4
5	N:ZnO (400 °C)	3.280	3.322	0.042	1.3
6	N:ZnO (500 °C)	3.270	3.305	0.035	1.1
7	N:ZnO (600 °C)	3.260	3.359	0.099	3.0
8	Al:ZnO (400 °C)	3.600	3.540	0.060	1.7
9	Al:ZnO (500 °C)	3.300	3.315	0.015	0.5
10	Al:ZnO (600 °C)	3.350	3.423	0.073	2.2
11	Cu:ZnO (as-deposited)	3.275	3.281	0.006	0.2
12	Cu:ZnO (400 °C)	3.260	3.280	0.020	0.6
13	Cu:ZnO (500 °C)	3.250	3.320	0.070	2.1
14	Cu:ZnO (600 °C)	3.258	3.308	0.050	1.5
15	Al/Cu:ZnO (as-deposited, 230 nm)	3.750	3.309	0.441	11.7
16	Al/Cu:ZnO (400 °C)	3.500	3.463	0.037	1.1
17	Al/Cu:ZnO (500 °C)	3.400	3.303	0.097	2.9
18	Al/Cu:ZnO (as-deposited, 50 nm)	3.800	3.519	0.281	7.4
19	Al/Cu:ZnO (as-deposited, 150 nm)	3.700	3.532	0.168	4.5

**Table 10:** Detailed quantum machine-learning predictions for Sample Set 2.

Index	Sample	Experimental (eV)	Predicted (eV)	Error (eV)	Error (%)
0	N:ZnO (300 °C)	3.300	3.176	0.124	3.7
1	N:ZnO (400 °C)	3.280	3.255	0.025	0.8
2	N:ZnO (500 °C)	3.270	3.260	0.010	0.3
3	N:ZnO (600 °C)	3.260	3.217	0.043	1.3
4	ZnO (as-deposited)	3.300	3.243	0.057	1.7
5	ZnO (400 °C)	3.260	3.231	0.029	0.9
6	ZnO (500 °C)	3.280	3.268	0.012	0.4
7	ZnO (600 °C)	3.260	3.278	0.018	0.5
8	Al:ZnO (400 °C)	3.600	3.445	0.155	4.3
9	Al:ZnO (500 °C)	3.300	3.170	0.130	3.9
10	Cu:ZnO (as-deposited)	3.275	3.296	0.021	0.6
11	Cu:ZnO (500 °C)	3.250	3.100	0.150	4.6
12	Al/Cu:ZnO (600 °C)	2.600	3.045	0.445	17.1

**Table 11:** Detailed quantum machine-learning predictions for Sample Set 3.

Index	Sample	Experimental (eV)	Predicted (eV)	Error (eV)	Error (%)
0	N:ZnO (300 °C)	3.300	3.330	0.030	0.9
1	N:ZnO (400 °C)	3.280	3.281	0.001	0.0
2	N:ZnO (500 °C)	3.270	3.269	0.001	0.0
3	N:ZnO (600 °C)	3.260	3.256	0.004	0.1
4	ZnO (as-deposited)	3.300	3.277	0.023	0.7
5	ZnO (400 °C)	3.260	3.259	0.001	0.0
6	ZnO (500 °C)	3.280	3.278	0.002	0.1
7	ZnO (600 °C)	3.260	3.255	0.005	0.2
8	Al:ZnO (500 °C)	3.400	3.334	0.066	1.9
9	Cu:ZnO (as-deposited)	3.270	3.300	0.030	0.9
10	Cu:ZnO (500 °C)	3.250	3.158	0.092	2.8
11	Al/Cu:ZnO (600 °C)	2.600	3.036	0.436	16.8

**Table 12:** Detailed quantum machine-learning predictions for Sample Set 4.

Index	Sample	Experimental (eV)	Predicted (eV)	Error (eV)	Error (%)
0	ZnO (as-deposited)	3.300	3.230	0.070	2.1
1	ZnO (400 °C)	3.260	3.263	0.003	0.1
2	ZnO (500 °C)	3.280	3.289	0.009	0.3
3	ZnO (600 °C)	3.260	3.247	0.013	0.4
4	N:ZnO (300 °C)	3.300	3.289	0.011	0.3
5	N:ZnO (400 °C)	3.280	3.286	0.006	0.2
6	N:ZnO (500 °C)	3.270	3.256	0.014	0.4
7	N:ZnO (600 °C)	3.260	3.248	0.012	0.4
8	Al:ZnO (500 °C)	3.300	3.283	0.017	0.5
9	Al:ZnO (600 °C)	3.350	3.258	0.092	2.7
10	Cu:ZnO (as-deposited)	3.275	3.232	0.043	1.3
11	Cu:ZnO (500 °C)	3.250	3.269	0.019	0.6
12	Al/Cu:ZnO (600 °C)	2.600	3.047	0.447	17.2

**Fig. 11:**
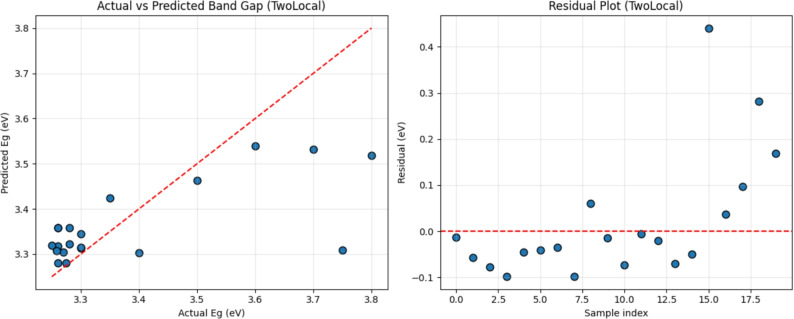
Actual (experimental) vs. Predicted band gap and residual plot for Dataset 1.

**Fig. 12:**
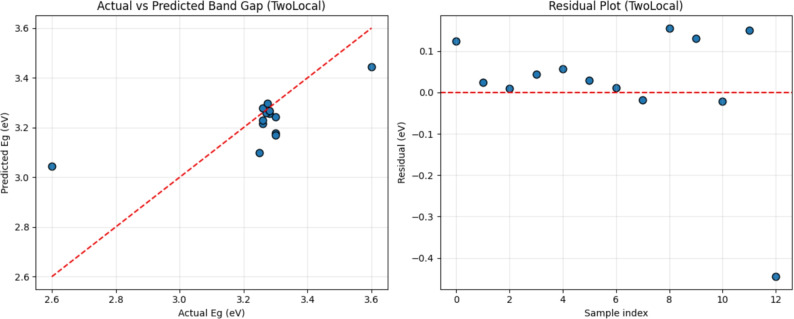
Actual (experimental) vs. Predicted band gap and residual plot for Dataset 2.

**Fig. 13:**
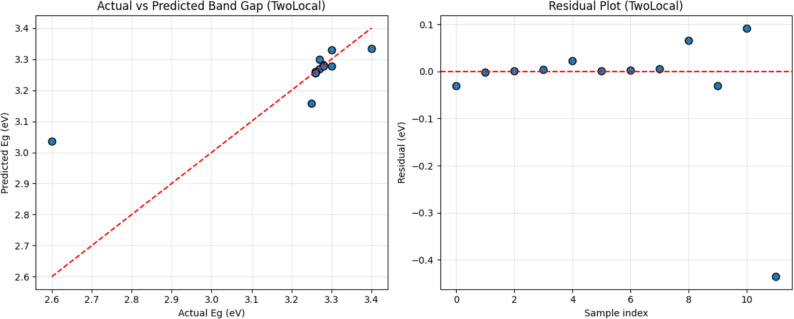
Actual (experimental) vs. Predicted band gap and residual plot for Dataset 3.

**Fig. 14:**
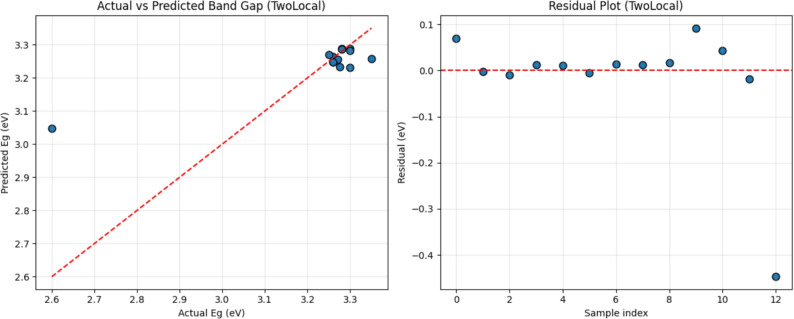
Actual (experimental) vs. Predicted band gap and residual plot for Dataset 4.

Figures 11, 12, 13 and 14 present the actual (experimental) versus predicted band gap values together with the corresponding residual distributions for all four datasets. Across all sample sets, the scatter points cluster near the ideal diagonal line for the majority of samples, indicating that the variational quantum regressor (VQR) successfully captures the primary trends governing the band gap behaviour in the typical range of 3.25–3.40 eV. The residual plots reinforce this observation, as most residuals lie close to zero for samples within the common band gap range. Dataset 3 exhibits the tightest clustering and smallest residual amplitude for the majority of samples, consistent with its low overall MAE and RMSE values, demonstrating that the combination of Urbach energy, RMS roughness, and lattice parameter provides highly complementary predictive information. Datasets 3 and 4 both contain one significant outlier sample with a band gap of 2.600 eV, which the models consistently underpredict (errors of 0.436 eV and 0.447 eV respectively), suggesting that the quantum model struggles with samples far outside the training distribution. Dataset 1 shows the widest spread in residuals, particularly for samples with high band gaps (3.75–3.80 eV), reflecting increased nonlinearity at the extremes of the band gap range. Dataset 2 displays moderate residuals for most samples but severe misprediction for the outlier sample, contributing to its highly negative test $R^{2}$. Overall, the four figures confirm that the VQR model exhibits consistent predictive capability for samples within the typical band gap range, with accuracy improving when the chosen descriptors carry strong and complementary correlations to the optical band gap, though outlier samples remain challenging for all feature combinations.

#### Quantum circuit structures used in the regression model

**Fig. 15:**
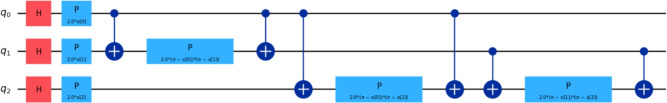
Decomposed ZZFeatureMap (reps=1, full entanglement) used for quantum feature encoding. This circuit embeds the classical input features via single-qubit *Z* rotations and *ZZ* entangling gates.

**Fig. 16:**
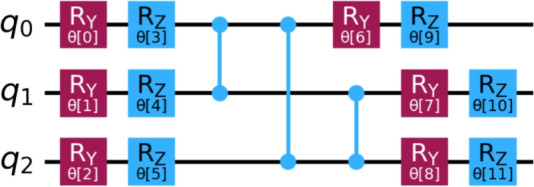
Decomposed TwoLocal ansatz (reps=1) composed of alternating $R_y$–$R_z$ rotation layers and controlled-*Z* entanglers, providing a variationally tunable quantum layer.

**Fig. 17:**
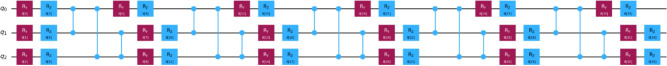
Full variational quantum circuit used in training, consisting of the ZZFeatureMap followed by a TwoLocal ansatz with five repetitions (reps=5). This deeper architecture enhances expressivity for learning nonlinear relationships in the dataset.

The circuit structures used in the quantum regression model are illustrated in Figs. 15, 16, 17. These figures show the progressive construction of the model, from feature encoding, to variational expressivity, to the final composite circuit used during training. Figure 15 displays the decomposed ZZFeatureMap (reps*=1*, full entanglement), which embeds the classical input features into quantum states using a combination of single-qubit *Z* rotations and pairwise *ZZ* entangling gates. This mapping introduces nonlinear correlations among the input variables, ensuring that feature information is distributed across all qubits. Figure 16 presents the decomposed TwoLocal ansatz (reps*=1*), consisting of alternating layers of parameterized $R_y$ and $R_z$ rotations followed by controlled-*Z* entangling operations in a fully connected topology. This variational template offers a compact yet expressive quantum layer capable of learning nonlinear transformations essential for regression. Finally, Fig. 17 shows the complete training circuit, where the feature map is followed by a deeper TwoLocal ansatz with five repetitions (reps*=5*). This substantially increases the expressive power of the model, allowing it to capture more complex dependencies between features and optical band gap values. Together, these circuits form the foundation of the Variational Quantum Regressor used in this study.

### Extended VQR validation and benchmarking for Sample Set 3

#### Effect of quantum circuit depth

The TwoLocal ansatz depth (number of repetition layers, *reps*) directly controls the number of trainable parameters and thus the model’s expressivity relative to the available training data. Table 13 summarizes the performance of VQR models with $\textit{reps} = 1, 2, 3,$ and 5 using an 80/20 train-test split.

**Table 13:** Effect of ansatz circuit depth on VQR performance in Sample Set 3.

Reps	Parameters	Param/N	Train MAE (eV)	Test MAE (eV)	Overall MAE (eV)	Overall $R^{2}$
1	12	1.33	0.065	0.093	0.072	0.472
2	18	2.00	0.063	0.095	0.071	0.495
3	24	2.67	0.050	0.076	0.057	0.536
5	36	4.00	0.060	0.051	0.058	0.541

All circuit depths yield overall MAE values between 0.057 and 0.072 eV, indicating that the model captures the dominant structure-property trends regardless of parameterization. The overall $R^{2}$ increases monotonically from 0.472 (*reps* *= 1*, 12 parameters) to 0.541 (*reps* *= 5*, 36 parameters), reflecting the improved expressivity of deeper circuits. However, the marginal improvement from *reps* *= 3* to *reps* *= 5* is modest (overall MAE: 0.057 vs. 0.058 eV; $R^{2}$: 0.536 vs. 0.541), suggesting that *reps* *= 3* (24 parameters, parameter-to-sample ratio of 2.67) provides a near-optimal balance between expressivity and parsimony. Notably, even the shallowest circuit (*reps* *= 1*, 12 parameters) achieves an overall MAE of 0.072 eV, which remains within the experimental uncertainty of Tauc-derived band gaps.

#### Regularization via early stopping

To assess whether limiting the number of optimizer iterations acts as an implicit regularization strategy, we trained the full VQR circuit (*reps* *= 5*, 36 parameters) with the L-BFGS-B maximum iteration count varied from 50 to 300. Table 14 presents the results.

**Table 14:** Effect of optimizer iteration limit (early stopping) on VQR performance in Sample Set 3.

Max Iterations	Train MAE (eV)	Test MAE (eV)	Overall MAE (eV)	Overall $R^{2}$
50	0.060	0.051	0.058	0.541
100	0.060	0.051	0.058	0.541
200	0.060	0.051	0.058	0.541
300	0.060	0.051	0.058	0.541

The results demonstrate that the L-BFGS-B optimizer converges rapidly for this problem, reaching a near-stationary solution within approximately 50 iterations. All metrics (training MAE, test MAE, overall MAE and $R^{2}$) remain essentially unchanged between 50 and 300 iterations (overall MAE *≈ 0.058* eV, overall $R^{2} \approx 0.541$). This rapid convergence suggests that the loss landscape is relatively smooth for this small dataset and that the optimizer does not explore substantially different regions of parameter space beyond the initial convergence phase. Limiting the optimizer to 50–100 iterations reduces computational cost by approximately a factor of three to five while preserving predictive accuracy; however, it does not act as an effective regularizer in our experiments, since the optimizer converges to essentially the same solution regardless of the iteration budget.

#### Classical baseline comparison

To contextualize the quantum model’s performance and evaluate whether a quantum advantage is achieved, we benchmarked the VQR against nine classical regression algorithms under identical conditions: the same 8-fold cross-validation protocol, per-fold feature scaling and evaluation metrics. The classical models span linear methods (Ridge, Lasso), kernel methods (Kernel Ridge Regression, SVR) and ensemble methods (Random Forest, Gradient Boosting). Results are presented in Table 15.

**Table 15:** Classical baseline comparison under 8-fold cross-validation in Sample Set 3 and out-of-fold predictions.

Model	8-fold cross-validation MAE (eV)	Non-outlier MAE (eV)
Ridge (*α = 1*)	0.141	0.071
Ridge (*α = 10*)	0.113	0.059
Ridge (*α = 0.1*)	0.167	0.093
Lasso (*α = 0.01*)	0.140	0.074
SVR (RBF)	0.116	0.062
Random Forest	0.086	0.031
Gradient Boosting	0.082	0.023
VQR (*reps* *= 5*)	0.091	0.056

Kernel Ridge Regression with RBF kernel (*γ = 0.5* and *γ = 1.0*) performed poorly on this dataset, likely due to overfitting with small *N*, and is omitted from the table for clarity. Several observations emerge from the classical comparison. Gradient Boosting achieves the lowest aggregated overall MAE (0.082 eV) and non-outlier MAE (0.023 eV), followed by Random Forest (0.086 eV overall, 0.031 eV non-outlier). The VQR model’s aggregated overall MAE (0.091 eV) is higher than the best ensemble methods. Importantly, the VQR model’s performance on the single 80/20 split (overall MAE *= 0.058* eV, Table 13) is substantially better than its 8-fold CV performance (0.091 eV), indicating sensitivity to the particular train-test partition. We conclude that the current VQR implementation does not demonstrate a clear quantum advantage over classical methods on this small dataset. Rather, the VQR framework should be understood as a proof-of-concept establishing the feasibility of variational quantum regression for materials property prediction. Potential quantum advantages may emerge with larger datasets, higher-dimensional feature spaces, or problems exhibiting quantum-native correlations that classical models cannot efficiently capture.

#### Comparison with experimental uncertainty

To assess the physical significance of the prediction errors, we compare the model MAE against the experimental uncertainty inherent in Tauc-derived optical band gaps. For sputtered ZnO thin films, the principal sources of uncertainty include the choice of linear fitting region in the Tauc extrapolation, baseline correction procedure, and film thickness measurement error. These factors collectively yield a typical uncertainty of *± 0.05*-0.10  eV^14, 17^. Table 16 provides a direct comparison.

**Table 16:** Comparison of model prediction errors with estimated experimental uncertainty in Tauc-derived band gaps.

Quantity	Value (eV)	Assessment
Experimental uncertainty in Tauc extrapolation		
Literature estimate^*a*^	$\pm \,0.05$-0.10	^14, 17^
VQR predictions (reps *= 5*, 80/20 split)		
Overall MAE (all 12 samples)	0.058	Within literature range
Non-outlier MAE (11 samples)	0.023	Below lower bound
VQR predictions (8-fold CV)		
Overall MAE (all 12 samples)	0.091	Within literature range
Non-outlier MAE (11 samples)	0.056	Within literature range
Best classical model (Gradient Boosting, 8-fold CV)		
Overall MAE (all 12 samples)	0.082	Within literature range
Non-outlier MAE (11 samples)	0.023	Below lower bound

^*a*^ Typical uncertainty range for Tauc-derived band gaps in sputtered ZnO thin films, arising from the choice of linear fitting region, baseline correction procedure, and film thickness measurement error.

For the 11 non-outlier samples, the VQR achieves a prediction error of 0.023 eV (80/20 split) to 0.056 eV (8-fold CV), both of which fall at or below the with the literature range $\pm \,0.05$-0.10. This indicates that, for samples within the dominant compositional range (undoped, N-doped, singly doped Al or Cu), the VQR predictions are comparable to the intrinsic noise floor of the experimental measurement itself.

#### Feature subset analysis

To evaluate the relative importance of the three structural descriptors and assess whether a reduced-dimensionality model offers improved generalization, we trained VQR models using all three possible 2-feature subsets (Table 17). Reducing from 3 to 2 qubits decreases the ansatz parameters from 36 to 24 (at *reps* *= 5*).

**Table 17:** VQR performance with 2-feature subsets compared with the full 3-feature model (80/20 split, *reps* *= 5*).

Feature pair	Parameters	Overall MAE (eV)	Overall $R^{2}$	Non-outlier MAE (eV)
$(E_{\textrm{U}},\textrm{RMS})$	24	0.078	0.446	0.044
$(E_{\textrm{U}},c)$	24	0.066	0.526	0.032
$(\textrm{RMS},c)$	24	0.081	0.462	0.048
$(E_{\textrm{U}},\;\textrm{RMS},\;c)$	36	0.058	0.541	0.023

The $(E_{\textrm{U}},\,c)$ pair yields the best 2-feature performance (MAE *= 0.066* eV, $R^{2} = 0.526$), approaching the full 3-feature model while using 12 fewer parameters. This result is physically intuitive: the Urbach energy captures the degree of electronic disorder introduced by doping and annealing, while the *c*-axis lattice parameter reflects the crystallographic strain and dopant incorporation. Together, these two descriptors encode complementary information about the electronic and structural state of the film. The RMS roughness, while correlated with microstructural evolution, appears to provide less independent predictive information for the optical band gap once $E_{\textrm{U}}$ and *c* are included. The full 3-feature model still achieves the lowest overall MAE (0.058 vs. 0.066 eV) and highest $R^{2}$ (0.541 vs. 0.526), indicating that the roughness descriptor does contribute incremental information, particularly for the outlier sample. For future studies with larger datasets, the $(E_{\textrm{U}},\,c)$ pair may provide a more parsimonious and robust starting point for quantum regression models.

**Fig. 18:**
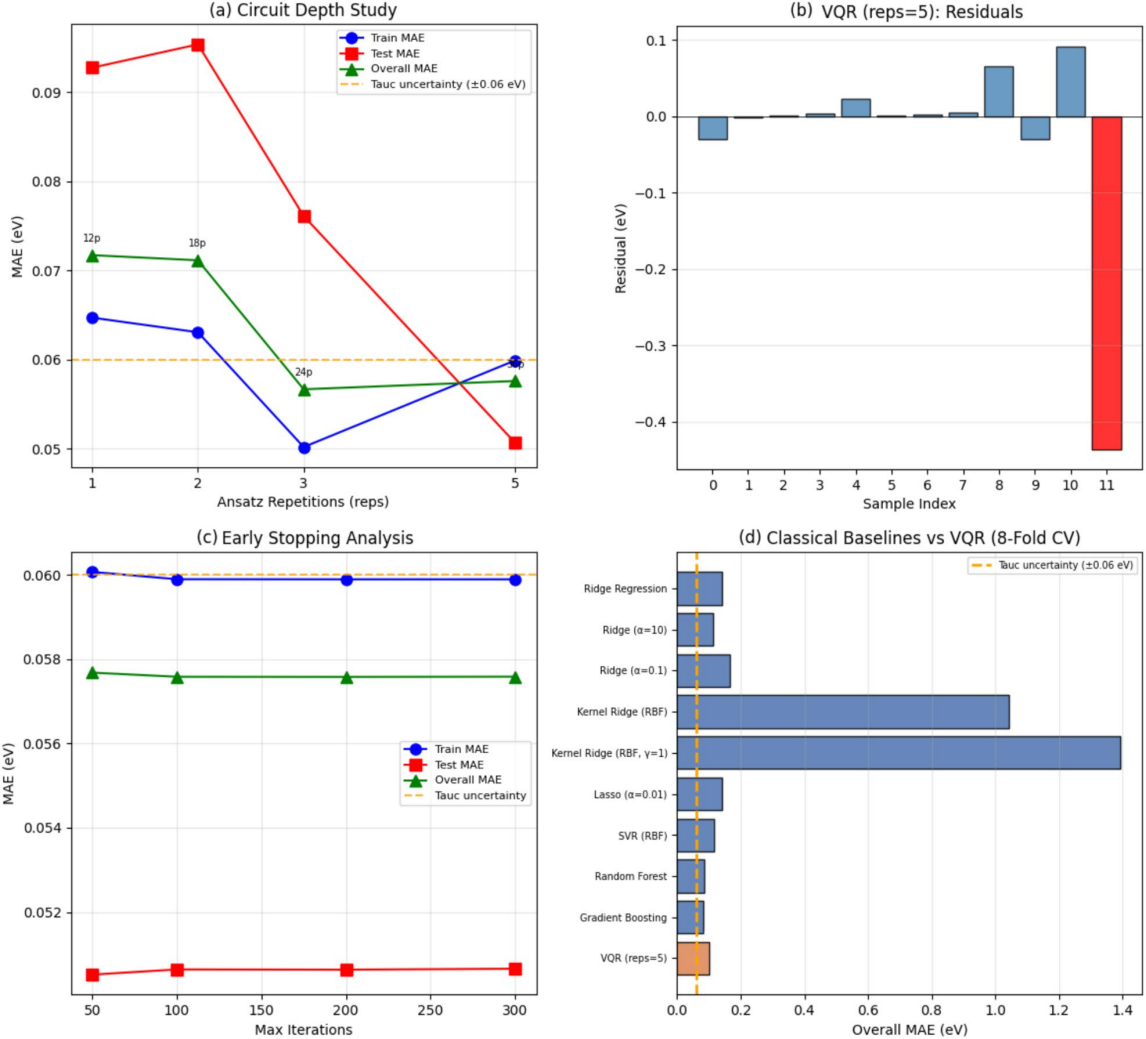
Composite diagnostic summary of VQR performance and comparison to classical baselines and experimental uncertainty for Sample 3.

At the end, to synthesize the findings from our supplementary analyses on Sample Set 3, Fig.  18 presents a comprehensive visual summary of the VQR model’s performance characteristics. This figure illustrates the impact of varying the ansatz circuit depth on the model’s predictive error, alongside a detailed residual analysis that clearly isolates the dominant error contribution from the anomalous Al/Cu:ZnO ($600^{\,\circ }$C) outlier. Additionally, it demonstrates the rapid convergence of the optimization process during the early stopping evaluation and visually benchmarks the VQR’s 8-fold cross-validation performance against a spectrum of classical regression techniques, placing the quantum algorithm’s current predictive capabilities into a broader methodological context.

### Clarification of the cross-validation and stratification strategy

Dopant identity is the dominant source of band-gap variation in our data: Al-doped films span 3.30-4.00 eV, the Al/Cu co-doped sample reaches 2.60 eV, while undoped and Cu/N-doped films cluster within 3.25-3.30 eV. If all samples of a given dopant class were to fall exclusively into the test partition of one fold, the model would have no training signal for that doping regime, producing misleading error estimates. The goal of the fold assignment was therefore to ensure that every fold’s training set contains at least one representative of each dopant category present in the dataset. Table 18 summarizes the number of samples per dopant category in each dataset.

**Table 18:** Number of samples per dopant category in each dataset. Singleton categories (1 sample) are shown in bold.

Category	Set 1 ($N{=}20$)	Set 2 ($N{=}13$)	Set 3 ($N{=}12$)	Set 4 ($N{=}13$)
ZnO	4	4	4	4
N:ZnO	4	4	4	4
Al:ZnO	3	2	**1**	2
Cu:ZnO	4	2	2	2
Al/Cu:ZnO	5	**1**	**1**	**1**

With *k = 5* folds, samples were distributed across the *k* folds according to the following rules:

(i) Categories with *\ge 2* samples (ZnO, N:ZnO, and depending on the dataset, Al:ZnO and Cu:ZnO) were spread across folds so that *at most one* sample from each category was held out in any given fold. This ensures that the training set of every fold contains at least one representative of these dopant classes. With only 2-4 samples per category, the test-set evaluation for each class in any single fold rests on at most one sample, limiting the statistical power of per-class test metrics.
(ii) Singleton categories were assigned to exactly one test fold and appeared in the training set for the remaining *k - 1* folds. In Sample Sets 2, 3, and 4, the Al/Cu:ZnO ($600^{\,\circ }$C) sample ($E_g = 2.60$ eV) is a singleton. In Sample Set 3, Al:ZnO ($500^{\,\circ }$C) is an additional singleton. These samples cannot be stratified: the model is trained without them in one fold and tested on them in exactly one fold. Consequently:
  The fold containing the Al/Cu:ZnO singleton in its test partition is the primary source of extreme negative test $R^{2}$, because this sample is simultaneously the most distributional outlier (0.65 eV below the next nearest) and incurs a prediction error of *∼*0.44 eV.
  The model has minimal training signal for the Al/Cu co-doped regime at high annealing temperatures, and no training signal at all for Al:ZnO in Set 3 during the one fold where it is held out. This is a fundamental limitation of the current dataset size.

The main results reported in the manuscript (Tables 9, 10, 11, 12) were obtained from a single 80/20 train-test split using random partitioning. Because each test set contains only 2-4 samples, ensuring per-class representation in the test partition is not always feasible when some categories have *łe 2* samples. For example, in Sample Set 3 the random 80/20 split places two Cu:ZnO samples and one N:ZnO sample in the test set, while ZnO (undoped), Al:ZnO, and Al/Cu:ZnO appear only in the training set. The supplementary *k*-fold CV analysis addresses this limitation by ensuring that every sample appears in a test fold exactly once, providing a more complete picture of generalization performance.

#### Remark 4.1

In the extended analysis of Sample Set 3, we additionally performed 8-fold cross-validation (*k = 8*) to provide near-complete out-of-fold coverage for *N = 12* samples (folds of 1-2 samples). This higher *k* was chosen specifically for the supplementary analyses because it maximizes the number of training samples per fold (10-11 out of 12) while still producing a full set of out-of-fold predictions. Both the original *k = 5* protocol and the supplementary *k = 8* results are reported; the latter yields an aggregated non-outlier MAE of 0.056 eV, consistent with the 80/20 split results (0.023 eV) and confirming that the model’s predictive accuracy for non-outlier samples is robust across validation schemes.

#### Remark 4.2

The datasets used in this study are small (*N=12*–20), which imposes concrete constraints on the interpretation of standard validation metrics. With 80/20 splits the test partitions contain only *2*–*4* samples, so the total sum of squares $\textrm{SS}_{\textrm{tot}}$ is estimated from very few points and can be vanishingly small when those points have similar targets. Because $R^{2}$ even modest absolute residuals may produce $\textrm{SS}_{\textrm{res}}>\textrm{SS}_{\textrm{tot}}$ and hence large negative per-fold $R^{2}$ values; such outcomes are a statistical artifact of tiny test sets rather than definitive evidence of uniformly poor predictive performance.

## Conclusion

Undoped, singly doped (Al, Cu, N), and co-doped (Al/Cu) ZnO thin films were deposited by reactive RF magnetron sputtering on quartz and systematically characterized after post-deposition annealing at 300–600 $^\circ$C. Thermal treatment promoted crystallite growth, strain relaxation, and improved crystallographic quality, as evidenced by XRD peak sharpening and shifts toward bulk ZnO lattice parameters. Cu and N dopants preserved the wurtzite structure, whereas high Al content caused partial amorphization in as-deposited films that recrystallized upon annealing at *\ge*400 $^\circ$C. All films exhibited continuous surfaces with RMS roughness between 0.94 and 9.67 nm, well below the film thickness (230–300 nm), Al produced the smoothest surfaces, N increased roughness at lower annealing temperatures and Cu yielded moderate, uniform grain morphologies.

Optical band gaps were tunable via dopant selection and annealing. Al doping produced Burstein Moss widening (up to *∼*4.0 eV), Cu doping caused slight narrowing (*≈*3.25–3.28 eV), and N doping left the gap close to the undoped value (*≈*3.26–3.30 eV). In general, annealing reduced the band gap through crystallite growth and reduced quantum confinement effects. Urbach energy analysis showed minimal band tail disorder in undoped ZnO (*∼*74.9 meV) and substantially increased disorder for Al doped and Al/Cu co-doped films (*≈*200–978 meV). Intermediate annealing (400–500 $^\circ$C) optimized crystallinity and minimized Urbach energy for most compositions.

Annealing at 500 $^\circ$C emerged as a critical processing window that balances crystallization, defect annealing, and dopant activation while avoiding the excessive grain coarsening and dopant redistribution observed at 600 $^\circ$C. The observed interplay between dopant chemistry, thermal history, and structural evolution demonstrates the versatility of doped ZnO thin films for targeted band-gap engineering.

Building on these experimental insights, we developed a quantum machine learning model that uses lattice parameter, Urbach energy and RMS roughness as primary descriptors. A variational quantum regression (VQR) implementation captured the nonlinear relationships between processing, structure, and optical response and achieved a best mean absolute error of **0.0576 eV** (overall MAE *≈* 0.058–0.094 eV across datasets), comparable to typical experimental uncertainty in band-gap extraction. While the proposed QML-assisted framework provides promising insight into the relationship between defects, optical properties, and bandgap engineering in ZnO thin films, the relatively limited dataset size (approximately 12-20 samples) may restrict the broader generalization of the model. In particular, the small number of experimental samples can increase susceptibility to overfitting and reduce the statistical representativeness of defect-related variations. Consequently, future work should include expanded datasets covering wider processing conditions and dopant concentrations, together with improved regularization and physics-informed learning strategies, to enhance model robustness and accelerate the rational design of ZnO-based transparent conductive oxides and advanced optoelectronic devices.

## Data Availability

All data supporting the findings of this study are available within the paper and its Supplementary Information.
